# Endocrine–Immune Crosstalk in Pediatric Autoimmune Diseases: A Systematic Review of the Role of Micronutrients, with Emphasis on Vitamin D

**DOI:** 10.3390/nu18193148

**Published:** 2026-09-24

**Authors:** Atapattu Navoda, Sunil J. Wimalawansa

**Affiliations:** 1 Department of Pediatric Endocrinology, Lady Ridgeway Hospital, Colombo 10, Sri Lanka; 2 CardioMetabolic & Endocrine Institute, North Brunswick, NJ 08902, USA

**Keywords:** 25(OH)D, 1,25(OH)_2_D, childhood, cost-effective, disease prevention, epidemiology, public health

## Abstract

**Background**: Pediatric autoimmune disorders are increasingly common, yet outcomes remain poor due to delayed diagnosis and lack of appropriate actions. Immune system dysfunction frequently coincides with deficiencies in essential micronutrients and cofactors, including vitamin D. **Objectives**: This study evaluated the contribution of vitamin D and cofactor (e.g., magnesium) deficiencies to impaired endocrine secretion and autoimmune susceptibility, which may trigger or exacerbate autoimmunity in children. **Methods**: This systematic review examines the complex interactions between micronutrients—particularly vitamin D—and cofactors such as zinc, selenium, probiotics, and omega-3 fatty acids, and their effects on endocrine and immune function. These interactions influence enzymatic activity, immune regulatory mechanisms, and endocrine pathways, potentially causing hormonal disequilibrium and increasing the risk of pediatric autoimmune disorders. **Results**: The review underscores the importance of early diagnosis, cost-effective non-pharmacological interventions, and targeted micronutrient strategies in reducing the risk, incidence, and severity of these disorders, prior to using expensive patented agents. Proactive supplementation—particularly with vitamin D_3_ and complementary cofactor micronutrients—known to strengthen immune functions, reduce inflammation and oxidative stress, and mitigate the development of autoimmune disease. Understanding the interconnected roles of micronutrient balance, gut microbiota, and immune dysregulation is essential for effective prevention and management of these conditions in childhood. **Conclusions**: This review encourages pediatricians and other healthcare professionals to adopt integrative, cost-efficient approaches—including holistic, orthomolecular strategies centered on personalized nutritional therapy—in addition to optimizing general health, to reduce the risk of autoimmune disorders and mitigate associated complications. Such approaches are likely to improve clinical outcomes, reform future research and public health strategy, and reduce healthcare costs. Optimizing micronutrient status may substantially enhance children’s well-being and long-term quality of life.

## 1. Introduction

The interactions between the endocrine and immune systems are crucial for maintaining health, particularly in pediatric autoimmune diseases. Throughout life, there is a significant interplay between the endocrine and immune systems. This relationship becomes critical during the period of rapid growth and development of the body, especially of the immune system. Autoimmunity occurs when there is maladaptation and the immune system erroneously targets and attacks the body’s own tissues, leading to chronic autoimmune diseases like type 1 diabetes mellitus (T1DM), Hashimoto’s thyroiditis, and adrenalitis, which are increasingly being diagnosed in pediatric populations [1]. While the cause of this escalation is unclear, as with immunosenescence in aging and comorbidities, endocrine and immune dysfunctions also increase vulnerability to autoimmune conditions during childhood.

The developing immune system in children continues to learn to distinguish self-antigen from non-self through antigen-sensing systems like Toll-like receptors, which remain active [2,3]. Disruption of this process can result in the immune system mounting an attack against the body’s own cells [4]. This vulnerability is enhanced by immune disruption that predominantly occurs when serum 25(OH)D concentration drops below critical levels, like 12 ng/mL—i.e., severe vitamin D deficiency when the immune system is paralyzed [5].

Many factors increase vulnerability to immune disruption, including environmental triggers (e.g., infections and pollutants), genetic predisposition, and deficiencies in key cofactors/micronutrients like vitamin D, magnesium, selenium, zinc, and omega-3 fatty acids [6]. They maintain physiological hormonal balance, hormone release, and receptor activity that prevent autoimmune tendencies [1,4]. Deficiencies in one or more of these nutrients could disrupt the immune and endocrine systems, heightening the risk of autoimmune diseases in children. Among these, vitamin D has the most prominent effect on regulating the immune system. Therefore, its deficiency is recognized as a key factor that increases vulnerability to autoimmune diseases [6]. This review examines the functions of micronutrients, particularly vitamin D’s interaction with immune and endocrine systems, focusing on pediatric autoimmune conditions.

### 1.1. The Endocrine System

The endocrine system comprises specialized glands, including the pituitary, thyroid, and adrenal glands, pancreas, ovaries, testes, and parathyroid glands. These secrete essential hormones that are involved in biological activities and physiological homeostasis, metabolism, reproduction, growth, and mineral balance. Calcitriol, produced by the distal renal tubular cells, functions as a hormone that regulates calcium homeostasis and musculoskeletal health. The parathyroid glands help maintain calcium homeostasis through calcium-sensing receptors [7]. It regulates intestinal calcium absorption, renal tubular reabsorption, and skeletal mineralization and demineralization [8].

In contrast, calcitriol produced in peripheral target cells (also modulated via tissue CYP24A1-mediated degradation)—which represents the quantitatively predominant source but does not enter the circulation—acts locally through non-endocrine mechanisms, including intracrine and paracrine signaling, rapid non-genomic signaling via membrane-associated pathways, and genomic activities [9,10]. Figure 1 summarizes the synthesis pathways, hormonal and non-hormonal actions, and key mechanisms of action of vitamin D.

Calcitriol has the highest affinity for its receptor and is present in most tissues and cells in the body, generating genomic functions. They are intricately involved in the regulation of hormones, like mineralocorticoids and glucocorticoids, and in their synthesis, activation, and release [13,14]. However, these need co-factors like magnesium, omega-3, and fatty acids [15,16]. In addition, essential antioxidants, trace elements, zinc, and selenium are required for many enzymatic reactions, thyroid function, and normal energy metabolism [17]. Knowledge of how these factors enhance the immune system has advanced significantly over the past few decades [18].

### 1.2. The Immune System

The immune system comprises innate and adaptive immune responses, which protect the body from pathogens and foreign microbial invaders [19,20]. Innate immunity is the first line of defense, providing a rapid response through mechanisms like phagocytosis and cytotoxic damage to pathogens [20]. In contrast, adaptive immunity is developed over time, in response to previous sensitizations. Consequently, the latter responses are more specific, involving the activation of B and T lymphocytes and memory cells for prompt and targeted responses based on previous encounters with allergens or pathogens [21]. The responses from adaptive immunity affect cells, tissues (e.g., lymph nodes), and organs like the spleen [20].

Immune regulatory mechanisms ensure T-cell and B-cell tolerance and prevent attacks on the body’s tissues. When tolerance fails, such as after acute viral infections, it can cause cellular and membrane damage, exposing native antigens that can cause an impairment of immune tolerance [22]. These can trigger an array of dysfunctional immune responses against self-antigens, initiating autoimmune disorders [23,24,25]. In response, the immune system produces pro-inflammatory cytokines, causing diffuse inflammation and oxidative stress—pathological reactions that can cause tissue damage [1]. In vulnerable individuals with comorbidities and low circulating 25(OH)D, it can also disrupt the hypothalamic–pituitary–thyroid (HPT) axis, increasing vulnerability. Vitamin D sufficiency prevents the immune system from becoming dysfunctional and hence reduces the risk of developing this negative vicious cycle of autoimmunity [26].

### 1.3. Interactions Between the Endocrine and Immune Systems

The endocrine and immune systems are interconnected, affecting each other through chemical molecules, receptors, and the paracrine system. Endocrine hormones modulate immunity, while immune cells release cytokines affecting endocrine functions. For example, cortisol, produced by the adrenal glands, modulates stress and inflammation [27]. With moderate responses, it prevents immune overactivation and tissue damage.

Therefore, to uncover disease mechanisms, progression, and focused treatments, it is necessary to understand endocrine–immune interactions in children. As discussed, severe vitamin D deficiency leads to major immune dysregulations, increasing susceptibility to infections, autoimmunity, and multi-system disorders [28]. The latter is a life-threatening autoimmune disorder [28] which includes multi-system inflammatory syndrome (MIS) and Kawasaki-like disease [29,30], as observed in children during the SARS-CoV-2 pandemic [30,31]. Early immune responses, including inflammation and oxidative stress, are natural physiological defense responses designed to overcome and eliminate infections, similar to the effects of rising body temperature during infection [32].

### 1.4. Common Pediatric Autoimmune Conditions

Autoimmune disorders occur when they harm cells or cellular components (i.e., within β-cells), causing inflammation, leading to tissue damage and ultimately organ dysfunction. Common autoimmune disorders in children include T1DM, juvenile inflammatory arthritis (JIA), systemic lupus erythematosus (SLE), celiac disease, Addison’s disease, and autoimmune thyroid disorders (AITD) [33,34]. These conditions arise from the immune system’s failure to distinguish self from non-self antigens, leading to autoantibody or autoreactive T-cell production that targets specific cell components or tissues.

### 1.5. Common Autoimmune Mechanisms

In addition to micronutrient deficiency and genetic susceptibility, environmental factors can contribute to the development of autoimmune diseases. Genetic predisposition, often involving the presence of various human leukocyte antigen (HLA) alleles, increases the likelihood of developing immune incompatibilities and autoimmune conditions [35]. However, genetic factors alone are typically insufficient to trigger disease onset without other risk factors, like exposure and/or micronutrient deficiencies [36]. Environmental factors, such as infections and exposure to toxins, can interact with epigenetics and enhance genetic risk, increasing susceptibility to immune dysfunctions [37].

In childhood, chronic hypovitaminosis D could be a triggering factor that interacts with environmental exposures and genetic susceptibility to increase autoimmune risks and trigger the pathogenesis of autoimmune diseases [36]. For example, in the presence of severe vitamin D deficiency, some viral infections can initiate an immune response against intracellular components that lead to autoimmune responses. Examples include pancreatic beta cell dysfunction precipitating T1DM in susceptible children [38,39]. Similarly, environmental factors like pollutants, excessive ultraviolet light exposure, and infections have been linked to the onset of SLE [40]. Another example is intra-uterine exposure to paracetamol in the presence of severe maternal hypovitaminosis D, which may increase the risk of autism spectrum disorders [41].

Critical reviews have addressed genetic [42] and environmental factors [43] related to complex interactions in developing autoimmune diseases [44,45]. These mechanisms provide evidence of their contributions through epidemiological studies and molecular and mechanistic research [46]. These studies highlight how genetic and environmental links and risk factors affect immune dysregulation [36,44]. In these cases, environmental exposures seem to act as catalysts for developing autoimmunity [36,43]. Understanding these mechanisms is crucial for developing strategies for prevention for individual patients, early detection, and targeted treatments [47,48] (see below for more information).

### 1.6. Endocrine Gland-Related Autoimmune Pathologies

Pediatric autoimmune disorders often disrupt both immune and endocrine functions. In AITDs, like Hashimoto’s thyroiditis and Graves’ disease, the immune system targets thyroid tissue, developing anti-thyroidal autoantibodies and leading to thyroid dysfunction and abnormal hormone levels. Hashimoto’s thyroiditis usually results in hypothyroidism, while Graves’ disease leads to hyperthyroidism [49]. Vitamin D deficiency is significantly associated with a higher risk of autoimmune diseases, both AITDs and T1DM [50,51].

In Type 1 diabetes, autoimmune destruction of pancreatic beta cells impairs insulin production, disrupting blood glucose regulation that requires lifelong insulin therapy. Similarly, in Addison’s disease, autoimmune adrenalitis damages the adrenal glands, reducing cortisol and aldosterone production essential for stress responses, electrolyte balance, and blood pressure regulation, requiring respective replacement therapies [1]. These diseases underscore the endocrine system’s susceptibility to immune-mediated damage, disrupting hormonal balance and causing lifelong health challenges [50,51]. Micronutrient cofactors, like vitamin D and magnesium [52], intensely affect the function of the endocrine and immune systems and the development of autoimmune disorders [52].

### 1.7. Restoring Balance: The Importance of Understanding Micronutrient Interactions to Alleviate Pediatric Disorders

Personalized medicine focuses on the use of micronutrients, nutraceuticals, functional foods, and orthomolecular medicine to restore the body’s optimal microenvironment (milieu) by correcting deficiencies and imbalances based on individual biochemistry and epigenetics. These approaches address health issues by restoring a healthy balance and targeting root causes using natural substances and micronutrients rather than prescription medications.

In addition to having clinical suspicion, understanding the complex interactions between the immune and endocrine systems is crucial for early detection of these disorders [53,54]. Once diagnosed, interventions focusing on maintaining a robust immune system using natural, functional foods and micronutrients (not expensive, patented medications) and avoidance of toxic products and exposure are essential and cost-effective ways to prevent autoimmune diseases [55].

Addressing root causes of autoimmunity, such as immune dysfunctions, severe vitamin D deficiency, and cofactor deficiencies, is key to restoring endocrine and immune balance and reducing autoimmune risks. This balance can be achieved with minimal costs by properly using micronutrients and cofactors—vitamins, minerals, trace elements, and essential fatty acids (see Section 2) [55]. Inherent vulnerabilities from hereditary or genetic defects and biochemical characteristics could be modulated by environmental pollutants [36]. In conjunction with physical and mental support, these factors are crucial for speedy recovery.

When managing such illnesses, biochemical pathways comprising enzymatic reactions are particularly important. Research indicates that identifying specific biochemical abnormalities can make the treatment more focused and cost-effective [54]. These abnormalities may respond to natural treatments and targeted supplementation. Advances in nutritional science, particularly personalized nutrition (which can be enhanced by AI), provide strategies based on metabolic profiles to prevent or slow down childhood autoimmune disorders [56]. Ensuring nutrient sufficiency through a balanced diet, food fortification, and targeted supplementation is a cost-effective way to improve children’s health outcomes [57].

## 2. Materials and Methods—The Study Review Process

This review examines the interactions between micronutrients, particularly vitamin D, and the endocrine and immune systems, and how deficiency can cause pediatric autoimmune disorders.

### 2.1. Systematic Review Process

This systematic review was conducted according to the Preferred Reporting Items for Systematic Reviews and Meta-Analyses (PRISMA) statement/guidelines using the PICOS (Participants; Intervention; Comparison/control; Outcome elements and Study design) criteria, as described below (Table 1) [58,59]. We followed guidance from the Equator Network (www.equator-network.org, accessed 2 April 2026), the PRISMA statement, and the PRISMA-P checklist [59,60] for assessing the relevance and quality of the literature. Data were gathered from pertinent scientific publications and critically analyzed and interpreted as described below.

### 2.2. Objectives of the Study

The objective of the study was to examine the role of micronutrients, particularly vitamin D, in endocrine–immune interactions and how they modulate risks of developing childhood autoimmune diseases. We evaluated prospective and retrospective analyses, case reports, and epidemiological studies, as well as randomized controlled clinical trials (RCTs), as illustrated in Table 1 [61,62,63].

### 2.3. Literature Search Strategy

The analysis is based on literature retrieved from PubMed, MEDLINE, EMBASE, Scopus, and the Cochrane Central Register of Controlled Trials databases. A systematic search was performed for original/primary studies, prospective and retrospective epidemiological and clinical studies, reviews, systematic reviews, and meta-analyses relevant to the topic [60]. Articles were included if they were focused on vitamin D as the primary supplement, vitamin D levels were measured, or discussed its mechanisms of action on autoimmunity and related endocrine and immune disorders, as well as related clinical outcomes. The literature search involved regular updates and full-text reviews [59]. Article selection was based on predefined autoimmune disease/disorders and vitamin D criteria.

Controlled search terms from Medical Subject Headings (MeSH) and the EMTREE thesaurus were used [58,59,64]. These include “vitamin D,” cholecalciferol, 25(OH)D, 25-hydroxycholecalciferol, calcifediol, calcitriol, childhood, pediatric, autoimmunity, autoimmune disorders. Some of these keywords were combined to refine, reduce, and manage search volume.

### 2.4. Protocol Eligibility Criteria: Inclusion-Exclusion and Manuscript Selection

The search followed the Preferred Reporting Items for Systematic Reviews and Meta-Analyses (PRISMA) statement and guidelines. It covered English-language articles published between January 1990 and May 2026. Selected studies were assessed thoroughly within conceptual frameworks aligned with the study’s objectives. Using a dual-keyword strategy, 199 duplicate records were removed, and the remaining 429 records were screened at the title and Abstract level. These were subjected to further screening for language, quality, and relevance, leading to the removal of an additional 73 records. After removing these, 356 articles were identified. An additional 48 relevant records were identified later via secondary and other sources. Full texts of 404 records were reviewed and included in this systematic review (SR) using EndNote 21.8 computer program, Philadelphia, PA, USA, Clarivate; 2023.

Evidence was prioritized based on relevance, methodological quality, and consistency. Owing to the multidisciplinary nature of the topic, a broad review approach was adopted [60]. Citations were screened for relevance, data were synthesized and categorized into subtopics, and hierarchical tables were created [59]. Additional evidence was drawn from peer-reviewed sources, web-based reports, and gray literature, which were scrutinized and added when peer-reviewed publication was not available; each was assessed for credibility, bias, and relevance, and rated appropriately [58,59]. Figure 2 outlines the systematic review process used.

### 2.5. Data Extraction—Abstraction and Synthesis

Data were extracted and synthesized using PRISMA guidelines as described above [58,65] and established methods described previously for a systematic review [58,59]. This subject covers pediatric autoimmune disorders, encompassing broader health research [60]. Sources and methodological quality were evaluated for alignment with the study topic and the aims, and findings were integrated into a coherent narrative, combining quantitative and qualitative evidence [60].

Assessments considered each study’s rationale, objectives, design, potential biases, and eligibility criteria. Data collection, analysis, and synthesis followed the evidence-based PICOS framework (Table 1) [65] and included meta-analytical approaches when applicable. Irrelevant and duplicate articles were excluded. The strength of evidence on vitamin D’s role in autoimmune disorders, incidence, mechanisms, clinical aspects, and outcomes was appraised critically. Results are documented as narrative conclusions [59]. Evaluations were integrated from observational studies and RCTs using the NIH National Heart, Lung, and Blood Institute quality assessment tools.

### 2.6. Outcome and Conclusions

In parallel with the global rise in vitamin D deficiency, the incidence of childhood autoimmune disorders has also been rising. Despite this, there is a lack of awareness among pediatricians, and significant gaps in knowledge exist. This includes using clinical suspicion, early identification, and interventions and implementation of cost-effective remedies, including balanced nutrition interventions to reduce risks for pediatric autoimmune disorders. Furthermore, recommendations on vitamin D and management guidance from governments and scientific societies are often contradictory and outdated, and guidance on sun exposure remains inconsistent [66,67].

Nevertheless, studies have consistently identified that long-term maintenance of serum 25(OH)D concentrations above 40 ng/mL [68,69,70] is the minimum effective level required to maintain a robust immune system. The optimal vitamin D intake needed for the maintenance of physiological therapeutic 25(OH)D blood level was identified using body weight or the BMI-tailored personalized calculation [32,68,69,71,72]. Considerations should include the costs and benefits of raising awareness across ethnic groups and populations and micronutrient cofactors to reduce autoimmune risks in the pediatric population [73].

### 2.7. Research Methods: Why RCTs Are Limited in Micronutrient Evaluations

There are several inherent limitations and challenges with RCT methodology when applied to micronutrients [74]. Importantly, micronutrients differ fundamentally from pharmaceuticals; therefore, drug-oriented RCT designs may not be optimal for evaluating nutrient effects [75,76]. These include but are not limited to subject recruitment issues, imprecise exposure measurement, analytical limitations, and difficulty establishing a true placebo condition [77].

Pharmaceuticals commonly have a low or absent baseline exposure and may show relatively linear dose–response relationships within the therapeutic range [78]. In contrast, micronutrients, including vitamin D, generally show “non-linear threshold responses”; benefit is greatest in individuals with deficiency or insufficiency and diminishes once physiological sufficiency is attained [68,78]. Consequently, trials that enroll participants with adequate baseline nutrient status (as with most recently published large vitamin D RCTs), use insufficient doses, fail to measure achieved blood 25(OH)D concentrations, or fail to detect meaningful separation in 25(OH)D concentrations between intervention and control groups underestimate or fail to detect a benefit [77,78,79,80].

As Heaney emphasized, the design and interpretation of nutrient trials should account for baseline nutrient status, dose, achieved biomarker concentration, co-nutrient interactions, and the threshold nature of nutrient responses [68,81]. Although RCTs remain indispensable for evaluating synthetic drug efficacy and safety and regulatory approvals, their application in nutrient research requires designs tailored to nutrient physiology rather than direct adoption of pharmaceutical trial models [74,78,80]. However, to date, this has failed to materialize. Growing reliance on conventional drug trial frameworks may partly reflect established research practices and funding structures, which do not always accommodate the complex and systemic roles of micronutrients in human health [78,82].

## 3. Role of Micronutrient Cofactors in Autoimmune and Endocrine Disorders

Micronutrient deficiencies, especially vitamin D, can initiate autoimmune conditions such as AITDs—Hashimoto’s thyroiditis and Graves’ disease—causing hypothyroidism and hyperthyroidism [83]. In T1DM, the resulting antibodies against cellular proteins continue to further destroy islet cells, reducing insulin synthesis and secretion, requiring lifelong insulin therapy [50,51], while autoimmune adrenalitis (Addison’s disease) impairs the ability to produce cortisol from the adrenal glands, causing life-threatening adrenal insufficiency [84]. These disorders highlight the close connection between autoimmune dysfunction and endocrine dysregulation.

### 3.1. Impact of Trace Minerals on Autoimmune and Endocrine Disorders

Autoimmune disorders can directly impact hormone production, causing hormonal imbalances as well as dysregulated immune responses [85]. For instance, SLE, in addition to immune dysregulation, can lead to endocrine issues like hypoadrenalism and hypothyroidism [86]. Besides, vitamin D deficiency and cofactor deficiencies can also cause tissue inflammation. Combined with the impairment of ACE-2 synthesis [87], hypovitaminosis D significantly disrupts the renin–angiotensin–aldosterone system/axis (RAAS), adding to this problem [87].

For example, following acute viral infections like SARS-CoV-2, overactive RAAS and suppression of ACE-2 [88] could lead to cytokine dysregulation, consequently increasing the risk of cytokine storms [87]. These also worsen endocrine abnormalities and autoimmune disorders. Understanding mechanisms related to autoimmune endocrinopathies is therefore essential for developing effective treatments and improving patients’ clinical outcomes.

### 3.2. Vitamins and Co-Factor Deficiencies Increase the Risks of Autoimmune Disorders

Chronic inflammation is a major contributor to autoimmune, metabolic, and cardiovascular disorders [55]. Micronutrients such as vitamin D, omega-3 fatty acids, and antioxidants, as described above, help regulate immune responses, suppress excessive inflammation, reduce oxidative stress, and may slow disease progression [84]. Future studies should investigate how these nutrient deficiencies influence excessive cytokine production, immune cell dysfunction, and oxidative stress [84], which are intricately associated with autoimmune diseases [89]. These might open the door for developing a novel class of immune therapeutics for preventing autoimmune disorders. In addition, a better understanding of mechanisms is likely to facilitate developing targeted nutritional strategies for preventing and managing pediatric autoimmune disorders [90].

The impact of micronutrients on the endocrine system is extensive. Maintaining cofactors plays a crucial role in hormone production, regulation, and secretion. They are essential for regulating endocrine functions as well as immune functions [50,51,84,91]. Their deficiencies contribute to conditions like hypothyroidism or adrenal insufficiency [92,93].

Deficiencies or imbalances of micronutrient intake affect the immune system and hormonal homeostasis and exacerbate inflammation and oxidative stress. They can also disrupt metabolic and overall immune functions, further increasing vulnerabilities [84]. Environmental pollutants can also aggravate existing diseases [94,95], including autoimmune disorders [12,96]. Table 2 outlines how vitamins and essential micronutrients influence immune activity, providing an overview of their role in immune and endocrine health.

Recent studies highlight how deficiencies in essential vitamins and minerals are also linked to increased risks and severity of autoimmune diseases (Table 1) [90,93]. These deficiencies are known to impair immune cell activity, weaken immune responses, and disrupt immune tolerance [125], as is the case with multiple sclerosis (MS) [86], rheumatoid arthritis (RA), and T1DM [43,126]. Similarly, zinc and selenium deficiencies can cause immune dysregulation, promote inflammation, and increase susceptibility to autoimmune disorders [84,93].

### 3.3. Polyunsaturated Fatty Acids: Modulators of Inflammation and Autoimmune Responses

Many chronic diseases, such as coronary artery disease, SLE, RA, and neurodegenerative disorders, are driven by underlying inflammation [127]. In addition to vitamin D (its most active metabolite, calcitriol), polyunsaturated fatty acids (PUFAs) [128], particularly the long-chain omega-3 PUFAs (*n*-3 LC-PUFAs), eicosapentaenoic acid (EPA), and docosahexaenoic acid (DHA) [129,130] possess significant anti-inflammatory and immunomodulatory properties [84,127].

Essential PUFAs and micronutrients help mitigate chronic inflammatory conditions, especially autoimmune disorders, including RA, Alzheimer’s, inflammatory bowel diseases, and SLE [130,131], and neurodegenerative disorders [84,131]. The anti-inflammatory effects of micronutrients promote protective cytokines and suppress inflammatory cytokines, thereby suppressing autoimmune diseases [91,129]. Mechanisms partly involve the modulation of inflammatory responses by reducing pro-inflammatory eicosanoids derived from omega-6 fatty acids. They also generate pro-resolving mediators such as resolvins and protectins. Consequently, in autoimmune conditions, they reduce inflammation and associated tissue damage [127]. These are some of the mechanisms by which receiving omega-3 supplements reduces the symptoms of joint pain and morning stiffness and reduces the reliance on non-steroidal anti-inflammatory drugs. These findings suggest that incorporating omega-3 fatty acids into the diet has a benefit as an adjunct therapy for managing autoimmune diseases [129,130].

Anti-inflammatory effects of omega-3 fatty acids reduce inflammation [132] and improve outcomes in autoimmune conditions such as T1DM [48,133,134]. Vitamins A, B, C, E, and K_2_, as well as minerals (zinc, iron, and selenium), have complementary benefits for immune health and endocrine functions (see Table 1). Integrating clinical judgment with advanced diagnostics, a multidisciplinary approach, and incorporating artificial intelligence (AI) would increase the accuracy and precision of diagnoses and improve the management of pediatric autoimmune diseases [135].

### 3.4. Recent Advances in Nutritional Immunology

Recent advances in this subject have highlighted the intricate link between diet, immune function, and health [136]. Immuno-metabolism explores how nutrients and metabolic processes shape immune cell responses and inflammation [137]. Understanding these interactions enables targeted nutritional interventions for effectively managing disease [138]. These emerging fields examine various aspects of immune regulation. An example is inefficient glucose utilization via aerobic glycolysis, which produces excess lactate in effector immune cells [139]. Additionally, studies on immune stress focus on how the immune system utilizes nutrition and metabolism to combat infections [136,137]. However, how these influence autoimmune disorders in childhood is unknown.

### 3.5. Examples of Complexities Associated with Childhood Autoimmune Disorders

HLA gene variants have been shown to increase genetic susceptibility to autoimmune T1DM [140] and JIA [141]. Notably, because HLA alleles modify risk and interact with non-HLA genetic and environmental factors, they do not determine whether a child will develop T1D or JIA but increase genetic susceptibility [142].

In genetically susceptible individuals, viral infections, cigarette smoking, and selected environmental chemical exposures may contribute to the initiation or progression of autoimmune–endocrine diseases. However, the strength and direction of these associations vary by disease and exposure [143], as in autoimmune AITDs. It discusses viral infections, cigarette smoking, radiation, iodine exposure, and chemical contaminants—including organochlorines, pesticides, polychlorinated biphenyls, and polybrominated diphenyl ethers—as relevant environmental factors in thyroid autoimmunity or thyroid dysfunction autoimmune thyroid diseases (AITD) [143].

Psychological stress, particularly when severe or persistent, has been associated with immune dysregulation and a higher subsequent risk of autoimmune disease; underlying genetic susceptibility is likely to modify individual risk [1]. It provides evidence that physical and/or psychological stress as possible triggers of autoimmunity, stress-hormone-associated immune dysregulation, cytokine changes, and reports that many patients retrospectively identify major emotional stress before symptom onset [1]. A Swedish nationwide cohort and sibling-comparison study found that individuals diagnosed with stress-related disorders after traumatic or severe stress had a higher subsequent risk of autoimmune disease than matched unexposed individuals and full siblings (adjusted HR, 1.36; 95% CI, 1.33–1.40) [144].

Dysregulated cytokine signaling, characterized by sustained elevations in pro-inflammatory mediators such as IL-6 and TNF-α and inadequate counter-regulatory activity of anti-inflammatory cytokines such as IL-10, can sustain inflammation and oxidative stress, thereby promoting conditions favorable to the initiation or progression of autoimmune disease [145]. This data supports persistent, dysregulated IL-6 production driving chronic inflammation, its involvement in a pathogenic role in autoimmune disease, and IL-6 as a key cytokine linking innate inflammation with adaptive immune responses [145,146].

Additionally, integrating multi-omics technologies—such as genomics, transcriptomics, proteomics, and metabolomics—may enable a more comprehensive understanding of how nutrition affects the immune system at the molecular level [136,138]. These approaches facilitate the identification of biomarkers and pathways that can be targeted through personalized nutrition strategies to enhance immune function and prevent disease [138].

### 3.6. Increasing Incidences of Environment-Induced Autoimmune–Endocrine Disorders

Research shows that combining polyphenols with essential micronutrients can suppress autoimmune activity and inhibit viral infections, such as those caused by influenza and SARS-CoV-2 [147,148,149,150]. Additionally, severe vitamin D deficiency (<12 ng/mL) has been linked to a 14-fold increased risk of harmful outcomes and higher mortality in SARS-CoV-2 infections, mostly mediated by immune dysfunction, highlighting the interactions and critical role of micronutrient status in immune and endocrine health [151].

## 4. Mechanisms—How Vitamin D Protects Against Pediatric Autoimmune Diseases

Vitamin D deficiency has become the most prevalent micronutrient deficiency in the world [76]. This is exacerbated by the increasing prevalence of indoor lifestyles, sun-avoidant behaviors, and urban air pollution [68,152]. Limited exposure to ultraviolet B (UVB) radiation, especially in temperate climates at higher latitudes, and darker skin pigmentation or skin damage (e.g., after burns) reduce the ability of the skin to synthesize vitamin D [153]. Fear of skin cancer from sun exposure and the widespread overuse of sunscreen and overclothing have increased the prevalence of vitamin D deficiency [68,154] and contribute to its global spread [76,155,156].

Over the past five decades, most countries have reported a gradual decline in mean serum 25(OH)D levels [157,158]. This trend parallels the rising incidence of obesity and type 2 diabetes, metabolic disorders, infections, sepsis [159,160] and autoimmune diseases [12]. Those with lower circulating vitamin D, and hence a dysfunctional immune system, were found to have more incidences of symptomatic disease and hospitalizations, increased complications, and greater mortality from SARS-CoV-2 [161,162]. Vitamin D-activating enzymes and VDR are expressed in all tissues. Vitamin D deficiency, through the mechanisms outlined below, has been shown to increase the risk of several diseases, including greater susceptibility to infections [159,160] and autoimmune disorders [12].

### 4.1. Definitions of Vitamin D Statuses

***Vitamin D Sufficiency****:* The current vitamin D guidelines and recommendations are based on the report from the Institute of Medicine (IoM), which is primarily focused on vitamin D’s effects on the skeleton and its role in the prevention of rickets and osteomalacia [163]. While the Endocrine Society (ES) Guidelines [164] were designed for individuals, the updated version of 2024 [153] reverts to the IoM foundation, focusing on skeletal benefits of vitamin D and ignoring [164] those in other body systems [165]. The IoM report is outdated [63,166]. In addition, the IoM report consists of faulty data [167], including major statistical errors that led to their Recommended Dietary Allowance (RDA) of vitamin D being ten-fold lower [163,167,168,169] for healthy people [170,171].

Many recent studies have shown that a physiologic range of 40–80 ng/mL (100 to 200 nmol/L; conversion factor: 1 ng/mL = 2.496 nmol/L) is the optimum for the populations [32,71] to effectively address metabolic disorders, infection, autoimmune diseases, and cancer. It covered an estimated 95% of disorders [76,77]. The minimum population 25(OH)D concentration to mitigate most disorders is considered 40 ng/mL [72,77,78], while for individuals, it is 50 ng/mL [32,71,172,173].

***Hypovitaminosis D:*** Hypovitaminosis D encompasses vitamin D deficiency and insufficiency—vague terminology referring to a serum 25(OH)D concentration less than 30 ng/mL (or 40 ng/mL based on the definition of sufficiency) [69,72]. These suboptimal circulatory 25(OH)D concentrations, which are inadequate for physiological functions of extra-skeletal tissues, are different than suboptimal levels for the musculoskeletal system [71]. 25(OH)D levels below 30–32 ng/mL are associated with increased circulating parathyroid hormone (PTH) concentrations [164,174,175], indicating that concentrations need to be higher for the extra-skeletal tissues to function properly. Hypovitaminosis D exacerbates most health disorders and increases susceptibility to infections and autoimmune disorders [12], illustrating the need for higher circulating D_3_ and 25(OH)D concentrations [68,176].

***Vitamin D Deficiency:*** Serum 25(OH)D concentrations below 20 ng/mL [75] are now considered indicative of vitamin D deficiency. At this level, vitamin D levels may still be sufficient to support calcium homeostasis and some essential musculoskeletal functions, including prevention of rickets in children and osteomalacia in adults, but may be inadequate for optimal function of other physiological systems in the body [75,76]. In addition to impaired immune function, such low vitamin D levels may contribute to the initiation or worsening of various disorders and increase susceptibility to multiple non-skeletal conditions [154,177]. Importantly, individuals with vitamin D deficiency generally respond well to appropriate supplementation, when indicated, with relevant cofactors such as magnesium, as discussed in Section 9.4. Consequently, this group of individuals represents an ideal candidate for recruitment into clinical trials [78].

***Severe Vitamin D Deficiency***: Severe vitamin D deficiency is a 25(OH)D concentration below 12 ng/mL; some studies use a cut-off value of 10 ng/mL. Individuals with severe deficiency may present with signs and symptoms of neuromuscular and skeletal dysfunction [76]. In addition, vitamin D deficiency worsens several diseases, increases complications and mortality, and causes premature deaths [68]. As Figure 3 summarizes, vitamin D deficiency causes a multitude of disorders, including increased risks for autoimmune diseases, and aggravates existing diseases.

Table 3 summarizes the extra musculoskeletal benefits of vitamin D, including diseases and disorders that have been shown to improve with long-term, adequate, and stable circulating 25(OH)D concentrations (as measured in serum).

### 4.2. The Way to Assess Vitamin D Status

The only reliable way to assess vitamin D status is to measure circulatory 25(OH)D concentrations [180]. There is no scientific evidence that the minimum adequate vitamin D levels vary across ages, latitude, ethnicity, or population groups [63,181]. However, the minimum effective (threshold) serum 25(OH)D concentrations vary based on tissues and diseases [12,71,75]. Peripheral target cells like immune cells do not have active transport systems across their membranes for D_3_ and 25(OH)D, so they rely on diffusion [182]. Consequently, diffusion of D_3_ and 25(OH)D into these target cells depends on maintaining higher circulating concentrations of them [32,71,183]. Based on these diffusion constants, recent data have substantiated the existence of tissue thresholds for D_3_ and 25(OH)D [77], such as for overcoming infections [71,172,173,184] and preventing autoimmune disorders [12].

In contrast, the IoM [185,186] and the ES guidelines (2024) [187] provide a minimum level of 20 ng/mL, which is based on the vitamin D requirements for the skeletal system (i.e., prevention of risks in children and osteomalacia in adults), and not for extraskeletal systems in vulnerable groups or sick people. Therefore, such recommendations are flawed and should not be used as clinical practice guidelines or for designing clinical studies related to extra-skeletal tissues, or policymaking [77,188].

### 4.3. Vitamin D Deficiency Enhances the Risks for Infections and Autoimmunity

Observational studies have shown that vitamin D reduces the risk of infections and autoimmunity, suggesting a causal link [176,189]. These studies have illustrated that a robust immune system [190] requires a minimum circulating 25(OH)D concentration of 50 ng/mL [173,191]. Despite this, most current guidelines focus on bone health while neglecting vitamin D’s broader roles in immune modulation and chronic disease prevention [75,192]. The current recommendations do not reflect a modern understanding of the systemic health impacts of vitamin D [77,78,193,194] and new vitamin D recommendations are required based on the latest data obtained from large, well-conducted observational studies [77,195]. For several reasons, RCTs are not the best way to evaluate any nutrient [78,176].

Ethnicity, genetics, skin, personal habits, and geographic location significantly affect vitamin D status [196]. Individuals with darker skin synthesize less vitamin D due to UVB rays being filtered by higher skin melanin content. Sun avoidance behavior in populations such as Asians and Middle Easterners, and those in other equatorial regions, increases the prevalence of hypovitaminosis D [75,197]. Vitamin D sufficiency prevents many chronic disorders and autoimmune conditions [12,68].

## 5. Consequences of Vitamin D Deficiency on the Immune System

As discussed above, sustained hypovitaminosis D causes immune dysfunction and susceptibility to chronic inflammation, infections, and an increased risk of autoimmune diseases [12,128]. The mechanisms involved include modulating innate and adaptive immunity, enhancing antimicrobial peptide production, generating neutralizing antibodies [64], reducing pro-inflammatory cytokines, and supporting regulatory T-cell function to prevent excessive immune activation [9,10,147].

Vitamin D deficiency impairs the functions of all immune cells, especially macrophage and dendritic cell functions, thus increasing the risk of intracellular bacterial infections like tuberculosis [198]. It also increases the risk of respiratory infections, such as influenza and SARS-CoV-2 [199,200]. Hypovitaminosis D (and associated cofactor deficiencies) can further increase autoimmune tendencies [201]. These can be mitigated by maintaining serum 25(OH)D concentration above 50 ng/mL [32,71] (as described in Section 8.2), which is feasible through daily sufficient sun exposure and/or vitamin D_3_ supplements [48].

### 5.1. Hypovitaminosis D Impairs Innate and Adaptive Immunity

Insufficient generation of calcitriol within immune cells [128] leads to a failure of activation of VDR/CTR, and consequently upregulates genes that synthesize, among others, antimicrobial peptides, like defensin and cathelicidin [182] that counteract microbes like Pseudomonas aeruginosa and others [202]. However, circulatory concentrations of calcitriol, even when externally administered (and diffuse at those very low levels into immune cells), are about 900-fold less than those of D_3_ and 25(OH)D, which are necessary to synthesize intracellular calcitriol [12,68].

While in vitro studies with very high pharmacologic doses of calcitriol have been shown to increase cathelicidin production in macrophages [103], such levels are unattainable and toxic in humans [68,154]. Consequently, calcitriol or its analogs should not be specifically used to prevent or treat infections or autoimmune conditions, as such very low concentrations fail to provide intracrine signaling or bind to VDR to stimulate DNA in immune cells (Figure 1) [12,76]. Instead, it is recommended to use higher doses of oral vitamin D_3_ (100–200 IU/kg) or, preferably, calcifediol (0.014 mg/kg), which has a rapid action, in such patients to overcome these disorders [32,71].

### 5.2. Vitamin D Deficiency Increases Susceptibility to Autoimmune Diseases

Vitamin D receptor (VDR) agonists have been used in the treatment of immune-mediated skin disorders such as psoriasis [203], and also used as topical therapies for this Th1/Th17-mediated inflammatory skin disease [204]. When calcitriol binds to the CTR, the resulting complex interacts with DNA, modulating approximately 2000 genes across various cell types, especially immune cells [205]. These genes are critical for maintaining immune tolerance and modifying responses associated with autoimmune disease risk [206]. These data underscore the significance of calcitriol and its cofactors in regulating immune responses and mitigating autoimmune conditions [206]. Figure 4 illustrates how vitamin D and its essential cofactors interact across the endocrine and immune systems and shows how deficiencies in these pathways increase physiological vulnerability and contribute to multiple comorbidities.

## 6. Activation of the Immune System Through Physiological Mechanisms

Vitamin D regulates immune cells, such as macrophages, dendritic cells, and monocytes [207], through genomic and non-genomic signaling [207,208,209,210]. However, most of these beneficial functions occur in response to the generation of calcitriol within immune cells [128,182]. This localized production allows immune cells to self-regulate, respond dynamically, and to sense pathogens and autoantigens “as needed” with appropriate immune responses [71,210]. Since their priming is dependent on intracellularly generated calcitriol, it is unsurprising that vitamin D deficiency (or cofactor deficiency) impairs immune cell functions, which increases the risk for autoimmune diseases, such as autoimmune T1DM and SLE [211,212,213,214,215], MS [216], and rheumatoid arthritis [217,218].

Hypovitaminosis D also impairs immune cells like T-helper cells, macrophages, and dendritic cell activation, the pathogen detection and defense system, and immune tolerance [210]. Meanwhile, the activation of T-helper-2 cells releases IL-10 (an anti-inflammatory cytokine), which dampens the innate responses and prevents the overactivation of the adaptive immune system, thus suppressing autoimmunity [219]. IL-10 also enhances pathogen clearance and ameliorates immunopathology [220].

### 6.1. Common Autoimmune Disorders Associated with Vitamin D Deficiency

Appropriate vitamin D supplementation has been shown to reduce the risk and improve clinical outcomes of autoimmune and inflammatory diseases [207]. Studies of MS, chronic fatigue syndrome, inflammatory bowel disease (IBD; Crohn’s disease and ulcerative colitis), and RA, etc., have demonstrated reductions in disease vulnerability, incidence, and severity following vitamin D supplementation [221,222,223].

However, randomized clinical trials (RCTs) have produced conflicting findings. These are primarily due to varied and poor study designs and too short a duration, such as using insufficient or too infrequent vitamin D dosing, neglecting cofactor deficiencies, and recruiting vitamin D-sufficient subjects to trials. Most RCTs failed to measure baseline serum 25(OH)D concentrations as a recruitment criterion, and did not measure the 25(OH)D levels achieved during the trials or correlate them with clinical outcomes [78]. Other confounders include taking over-the-counter multivitamin preparations, individual variability in responses, achieved serum levels after supplementation with the same doses, and genetic polymorphisms (e.g., GC, CYP2R1, CYP24A1, and VDR variants) affecting vitamin D metabolism and signaling [211,212,213,214,215].

It is critical to consider both the administered vitamin D (or calcifediol) dose and the resulting 25(OH)D concentration—the marker of sufficiency—in clinical studies and practice, since different tissues have distinct thresholds for optimal immune response [207,224,225]. Moreover, suppressing inflammation and enhancing immune tolerance may offer further therapeutic potential in autoimmune diseases. Further research could clarify how to optimize immune function through sustainable lifestyle changes and micronutrients, improving patient health [226].

### 6.2. Immune Cell Activation by Vitamin D

Evidence shows a robust inverse relationship between 25(OH)D levels, cytokine generation, and autoimmunity [46]. Low serum 25(OH)D concentrations increase the risk and severity of autoimmune diseases [227]. Figure 3 highlights the adverse outcomes of chronic vitamin D deficiency.

Immune activation occurs through tightly regulated mechanisms that detect, respond to, and eliminate harmful stimuli while preserving tissue balance and avoiding damage to normal cells. These responses depend on coordinated interactions among innate and adaptive immune cells, signaling molecules, and tissue-specific pathways that support host defense, repair, and immune surveillance. Understanding these mechanisms helps explain how normal functioning immunity protects against infection and how dysregulation contributes to inflammatory and autoimmune disease [12].

### 6.3. The Importance of Maintaining Physiological Levels of 25(OH)D

As Section 4.1 explains, circulating calcitriol is too low to enter most cells efficiently. Renal tubular, bone, parathyroid, and muscle cells internalize DBP-bound vitamin D metabolites, including calcitriol, through megalin–cubilin transport, but most peripheral target cells lack this system [228]. In addition, 25(OH)D_3_ complexed to vitamin D-binding protein (DBP) filtered at the glomerulus is reabsorbed by renal proximal-tubule cells via the endocytic receptor megalin [228].

Thus, immune and other peripheral cells rely mainly on circulating vitamin D_3_ and 25(OH)D to synthesize intracellular calcitriol [33,87]. This explains why pharmacologic calcitriol or analogs offer limited benefit for these cells and may cause adverse effects. It also supports maintaining steady vitamin D_3_ and 25(OH)D levels, preferably through daily rather than intermittent vitamin D administration.

Monocytes, macrophages, neutrophils, hematopoietic stem cells, and activated T and B cells express the enzymes CYP2R1 and CYP27B1 hydroxylase, which convert D to 25(OH)D [229,230] and 25(OH)D to its most active form, calcitriol [231,232]. Immune cells also actively synthesize CTRs as needed, with their expression regulated by activation signals from distantly located signaling cells [233]. Activation of T-cells (and other immune cells) triggers acute increases in CTR expression in response to external activation signals from other immune cells and the cytokine network [234], via genomic activation [235].

Calcitriol and CTR expression in immune cells occur in response to demand, based on signals they receive from outside—an essential evolutionary biological step [236]. Activation of 1α-hydroxylase within immune cells depends on signals received through antigen-recognition pathways such as Toll-like receptor-4 (TLR4) [2,237], enabling cyclical, on-demand generation of calcitriol [236]. Activating B- and T- immune cells is also dependent on the locally produced calcitriol within them [236,238]. This process becomes less robust during vitamin D deficiency, thus impairing immune cell activation [234].

These genomic activations, which are secondary to signaling from pathogen and foreign antigen detection cells, lead to the production of antimicrobial peptides, such as cathelicidin and β-defensin 2. Optimization of the responses from monocytes, macrophages, and T-cells occurs through micronutrients, especially calcitriol, which utilizes its intracrine and genomic signaling (Figure 1; see next section) [12,77,78,177]. This boosts their anti-microbial and protective activities [207].

This emphasizes the importance of maintaining adequate circulatory vitamin D and 25(OH)D concentrations for a robust immune system, which is also crucial for preventing autoimmune disorders [12]. It also explains the rationale for taking vitamin D daily or once a week, rather than at a lower frequency, like monthly [77,78,239,240]. This cascade of physiological actions is known to reduce risk and susceptibility to infections and autoimmunity and mitigate the severity of acute and chronic conditions [46,241,242]. The opposite occurs in vitamin D deficiency.

### 6.4. Intracrine and Paracrine Signaling System

Calcitriol, synthesized within peripheral target cells, plays a critical role in intracellular signaling (intracrine/autocrine, paracrine, and genomic functions) [9,10,71]. For example, intracrine signaling of calcitriol switches T helper cell 1 (Th1) to T helper cell 2 (Th2) and Th17 to Treg cells [219], transforming them from a pro-inflammatory status to an anti-inflammatory status [243,244]. The failure of this switching maintains the inflammatory status of Th1 and Th17 cells (as in chronic hypovitaminosis D) [32]. This is one of the key mechanisms in vulnerable individuals following severe viral infections, such as SARS-CoV-2, which can initiate cytokine storms and the development of ARDS [245,246].

Induction of CTR and 1α-hydroxylase (from the *CYP27B1* gene) within immune cells initiates critical anti-inflammatory responses and Th1 responses, allowing their transition from a pro-inflammatory interferon-γ+ status to Th2 cells that secrete interleukin-10 [247]. This process facilitates changes in CD4+ T cells and T-regulatory transcription factors [248], such as c-JUN, BACH2, and STAT3, modulating CTR transcription [249], whereas vitamin D deficiency dampens the expression of interleukin-10 and Th1 cells [140], leaving them in inflammatory status. Consequently, insufficient vitamin D leads to sustained hyper-inflammatory status in severe infections like SARS-CoV-2, increasing complications and deaths [250].

### 6.5. Vitamin D: A Key Modulator of Intracellular Signaling

When calcitriol synthesis is required, expression of the converting enzymes CYP2R1 and CYP27B1 rapidly increases within immune cells to hydroxylate D_3_ and 25(OH)D into calcitriol [234,251]. As intracellular D_3_ and 25(OH)D are consumed, more diffuses in from the circulation (this becomes easier when the blood levels are maintained above 50 ng/mL) [32,71]. The newly synthesized calcitriol then engages in autocrine and paracrine signaling and genomic activation. This cycle stops once the external signal (e.g., from Toll-like cells)—the microbial threat—resolves [252,253].

These data show that higher circulating 25(OH)D concentrations upregulate CYP27B1 expression in antigen-presenting cells, promoting local calcitriol synthesis and confirming diffusion-dependent regulation of immune function [251]. This also supports the rationale that D_3_ and 25(OH)D availability—reinforcing the need for daily supplementation or sun exposure rather than infrequent dosing—regulates immune cell function [251].

A 25(OH)D concentration above 50 ng/mL is sufficient for robust immune cell functions, including mitigating autoimmunity [12] and infections [159,160]. At these blood concentrations, inflammatory T-helper cell 1 (Th1) responses are shifted to anti-inflammatory T-helper cell 2 (Th2), and T-helper cell 17 (Th17) responses to regulatory T (Treg) cells [254]. The transition of Th1 to Th2 is vital to maintain an anti-inflammatory environment [243,244]. In addition, vitamin D is also critical for Toll-like receptor signaling and related immune surveillance against infections and autoimmune/inflammatory diseases [255]. Like other immune cells, T cells also have CTRs, activated through locally produced calcitriol [128,182].

## 7. Mechanisms of Vitamin D Modulating Childhood Autoimmune Disorders

Pediatric autoimmune diseases often result from interactions among micronutrient deficiencies, environmental factors, infections, and immune dysregulation in genetically predisposed children. Among these factors, vitamin D deficiency plays a predominant part in increasing susceptibility. Interactions of vitamin D metabolites with immune cells were described in Section 5 above. This section focuses on underlying mechanisms that are necessary to understand and develop strategies to mitigate autoimmune diseases.

### 7.1. How Vitamin D Mitigates Autoimmune Disorders

The mechanisms of action of vitamin D are complex and involve genomic functions via CTR [256,257,258,259] and the membrane [243,244,260,261], as well as intracrine/paracrine signaling pathways in target cells [32,243,244,260,262,263]. Vitamin D protects against tissue and DNA damage caused by overactive T-cells and prevents the development of autoimmune reactions [264,265,266]. As detailed in Section 5, calcitriol synthesized within immune cells enhances anti-inflammatory processes and suppresses hyper-inflammatory responses [160]. In addition, it re-establishes the balance between T cells, promoting immune tolerance [259,267,268]. In contrast, vitamin D deficiency increases the risk of initiating and worsening autoimmune diseases like MS [216], RA, and T1DM [177,269,270,271,272]. Therefore, it is unsurprising that proper vitamin D supplementation mitigates the progression of autoimmune diseases.

### 7.2. Vitamin D Deficiency Aggravates Childhood Autoimmune Disorders

Chronic inflammation is a hallmark of autoimmune disorders. Breakthrough acute exacerbations occur in many pediatric autoimmune disorders that affect vital organs and substantially impair quality of life. For example, T1DM results from the autoimmune destruction of pancreatic β-cells, leading to insulin deficiency and hyperglycemia [273,274]. JIA is characterized by persistent joint inflammation, causing pain, swelling, and progressive joint damage [275]. AITDs, including Hashimoto’s thyroiditis and Graves’ disease, target the thyroid gland, resulting in hypothyroidism or hyperthyroidism [276,277].

Celiac disease, although less common, is also an immune-mediated disorder triggered by gluten that damages the intestinal mucosa, leading to malabsorption and gastrointestinal manifestations [275,278]. Early diagnosis and appropriate management of these autoimmune disorders are essential to mitigate the disease and prevent long-term complications. Hypovitaminosis D, by impairing immune regulation, not only precipitates but also exacerbates these conditions. Figure 5 summarizes the multitude of effects of vitamin D, which crosstalk with endocrine and immune cells, leading to the development of childhood autoimmune disorders.

## 8. Hypovitaminosis Aggravates Childhood Autoimmune Disorders

Lack of clinical suspicion, incomplete clinical evaluations, and/or inadequate laboratory testing could lead to misdiagnosis, underdiagnosis, or delayed diagnosis of pediatric autoimmune diseases [279]. Key diagnostic tools include using specific autoantibodies indicative of cell or tissue destruction, assessing hormonal levels to evaluate endocrine gland function, and utilizing imaging studies to clarify tissue damage [280]. Table 4 summarizes examples of the major pediatric autoimmune disorders, their pathological features, and the complex interplay between genetic and environmental factors, highlighting the need for early diagnosis and targeted preventive and therapeutic interventions. Additional examples of pediatric autoimmune disorders are described below.

### 8.1. Type 1 Diabetes Mellitus (T1DM)

Type 1 diabetes mellitus (T1DM) results from the autoimmune-mediated destruction of insulin-producing pancreatic β-cells [284], causing insulin deficiency [285]. It is one of the most clinically significant pediatric endocrine diseases, and its incidence is increasing globally. Recent studies have highlighted several early-life factors that influence the development of T1DM in children [285,286]. Infants born by cesarean section and those not breastfed or breastfed for a shorter duration, and infants of mothers with hypovitaminosis D, have a higher likelihood of developing T1DM later in life [285]. This suggests effects of these on early immune system development and possible microbiome exposure in disease onset [287,288].

In addition to genetic factors and viral infections, exposure to unhealthful environmental factors and micronutrient deficiencies during early childhood increases susceptibility to autoimmune diseases like T1DM, as well as autism [36,289]. During the COVID-19 pandemic, a notable rise in T1DM among children was observed [255]. However, the exact interplay between them remains complex and not well understood [286,290]. Early life healthy interventions, such as breastfeeding, are reported to have a protective effect, reducing the incidence of T1DM [289]. However, whether these benefits are due to sufficient micronutrients, modulating immune responses, better healthcare, minimizing harmful exposure, or fostering gut microbiota balance is unclear [290].

Several ongoing studies are exploring the role of vitamin D in respiratory infections [291], halting the progression of T1DM [278], and improving islet transplant survival [292]. Vitamin D sufficiency is vital for genes controlling insulin secretion and its functions [293]. Others have reported that vitamin D supplementation and/or exposure to UVB rays reduce the incidence of T1DM in children [294,295]. Evidence suggests that seasonal variations (e.g., winter) and geographic regions (i.e., higher latitudes, with less sun exposure and colder climates) are associated with a higher prevalence of vitamin D deficiency and an increased incidence of T1DM. These data support the hypothesis of the role of vitamin D in preserving β-cells [283] through beneficial, immunomodulatory effects [134,274,284].

Other researchers have reported that children migrating from low socio-economic regions to higher socio-economic areas had an increased tendency to develop T1DM [296], suggesting the possibility that other environmental factors, such as diet, lifestyle, and exposure to different infections, trigger the autoimmune destruction of β-cells in susceptible individuals. Studies have also linked these environmental shifts to altered immune regulation and increased autoimmune responses, contributing to the onset of T1DM [284,297,298]. These findings point out the complex interplay between genetics [6] and environmental factors contributing to the development of autoimmune diseases like T1DM [95].

### 8.2. Autoimmune Thyroid Disease (AITD)

Autoimmune thyroid diseases (AITDs) include Hashimoto’s thyroiditis and Graves’ disease, with Hashimoto’s being the most common cause of acquired immune-mediated hypothyroidism in pediatric patients [299]. The disease is rare in children under three, with peak incidence occurring during the pubertal years and has a female preponderance [300]. The pathogenesis of Hashimoto’s thyroiditis involves the immune system targeting specific thyroid autoantigens, including thyroglobulin (Tg) [299] and thyroid peroxidase (TPO) antibodies, and the development of antibodies against the thyrotropin receptor (TSHR). These autoantibodies play a significant role in the destruction of the thyroid gland, leading to hypothyroidism. Their early detection is vital for diagnosing and understanding the disease’s progression and for preventing it from affecting the child’s growth [301].

Thyroid autoantibodies can also be detected in children with a family history of AITD [302]. Chronic hypovitaminosis D increases the risk of developing AITDs, highlighting the critical role of adequate vitamin D status in mitigating AITDs [51]. Identification of circulating autoantibodies, like TPO and anti-thyroglobulin antibodies (TgAb), is strongly associated with the later onset of hypo- or hyperthyroidism [302]. As discussed below, hypovitaminosis D is strongly linked to autoimmune thyroiditis in pediatric and adult populations, highlighting its role in thyroid health [47,272].

A rising prevalence of AITD was reported during the COVID-19 pandemic, which further supports that viral infections can trigger autoimmune responses in vulnerable children [303,304]. While this is not a new phenomenon, viral infections have been recognized to trigger autoimmune conditions such as Hashimoto’s thyroiditis and Graves’ disease for many years [305]. Additionally, growing evidence highlights the role of gut microbiota imbalance in contributing to the development of AITD [126,269].

In this regard, synbiotic supplementation (a combination of prebiotics and probiotics) has been shown to reduce TSH levels and decrease the required dose of thyroxine in patients with autoimmune hypothyroidism [306]. Furthermore, medications, such as amiodarone, can cause chemically induced thyroiditis that could lead to autoimmune thyroiditis, underscoring the vigilance needed for pharmacological agents in thyroid autoimmunity [46]. Children with anti-TPO or anti-Tg antibodies (even with normal thyroid function) are at a higher risk of developing hypothyroidism secondary to drugs like amiodarone and sustained hypovitaminosis D, highlighting the complex interplay between genetic, environmental, and pharmacological factors in AITD [307].

### 8.3. Autoimmune Impact on T4 Conversion

Higher levels of thyroid autoimmune antibodies can disrupt the conversion of T4 to active T3 via deiodinases. The biologically active hormone, T3, eventually binds to intracellular TH receptors (TRs), TRalpha and TRbeta, that initiate TH signaling, regulating target genes and metabolic pathways. Alternatively, T4 can be deactivated (i.e., T3 to 3,3′-diiodo-l-thyronine or T4 to reverse T3 conversion) by excess presence of T3 or autoimmune antibodies, which favors reverse T3 (rT3) production that blocks T3 receptors. This presents as resistance status [308,309] or low T3 syndrome [310]. This imbalance may result in persistent hypothyroid symptoms despite normal thyroid hormone levels.

T3 augmentation can address this issue by bypassing the T4-to-rT3 conversion, directly reducing TSH and T4 production, and limiting rT3 formation, as in the case of Hashimoto’s thyroiditis [311]. However, T3 is more potent but has a significantly shorter half-life than T4. T3 supplementation significantly impacts TSH levels and biological actions, thus requiring careful monitoring to avoid adverse effects [312]. In these cases, daily divided doses of T3 are administered to maintain consistent therapeutic effects while preventing adverse effects.

### 8.4. Autoimmune Addison’s Disease

Adrenal insufficiency, characterized by impaired secretion of glucocorticoids and mineralocorticoids, can be a life-threatening condition. It can be congenital or acquired, with autoimmune adrenal insufficiency (Addison’s disease) being the most common cause of acquired adrenal insufficiency in younger patients [313]. This occurs when autoantibodies target three zones of the adrenal cortex.

Genetic factors, such as the HLA DRB1 gene variant, are a common risk factor for developing Addison’s disease [35]. Epigenetic modifications can further elevate the risk of autoimmune disease by modulating gene expression and transcription [46,314]. Understanding these mechanisms is critical for advancing the development of targeted therapies for autoimmune diseases in childhood [313].

## 9. Core Strategies in Preventing Pediatric Autoimmune Diseases

Preventing pediatric autoimmune diseases requires, as discussed above, an early-life multifaceted strategy focused on immune health, environmental risk reduction, and timely clinical intervention. Although many autoimmune conditions have a strong genetic basis/epigenetics, onset and progression can be influenced by modifiable factors such as infections, nutrition, microbiome development, obesity, and exposure to environmental triggers. Authors highlight the importance of a prevention-oriented approach. Therefore, promoting healthy childhood development, early risk identification, and early supportive care can reduce the likelihood of having and/or the severity of autoimmune disease over time.

### 9.1. Core Treatment Strategies for Pediatric Autoimmune Disorders

As with many other disorders, the current approach to managing pediatric autoimmune diseases focuses on symptom management, inflammation control, and progression prevention, with disease prevention somewhat neglected. For example, vitamin D and omega-3 fatty acid supplementation [48,73] have been shown to have potential benefits in reducing the risk of T1DM [133,134] and have less impact on other autoimmune diseases in childhood. The benefits of vitamin D are derived from modulating immune functions, enhancing immune tolerance, and reducing autoimmune-mediated destruction (e.g., β-cells) [133,134]. Collectively, these actions slow the progression of T1DM [132,315]. Further clinical research is needed to elucidate fundamental mechanisms and develop agents (or repurpose approved drugs) that are economical to use, focused, and have fewer adverse effects in children.

In addition to vitamin D and omega-3 fatty acid supplementation, other dietary strategies, such as reducing intakes of arachidonic acid-containing foods and following a Mediterranean diet, have also been investigated [16,316]. Combination immunotherapy in type 1 diabetes is being explored as a strategy to pair early anti-inflammatory or immune-modifying treatment—aimed at limiting islet inflammation—with approaches intended to restore immune tolerance and preserve residual β-cell function [317]. Although this approach has a sound biological rationale and may offer potential benefit, evidence for sustained β-cell preservation remains limited, and durable clinical remission has not been consistently demonstrated in human trials [317]. Meanwhile, combination therapy in autoimmune T1DM aims to limit islet inflammation early while restoring immune tolerance and preserving β-cell function. However, no single therapy had achieved durable remission, and early combination trials showed limited or disappointing results [318].

A combination is likely more beneficial than treating with individual components or a single medication. However, the long-term effectiveness of the current regimens remains uncertain, emphasizing the need for rigorous clinical trials to confirm their clinical impact on pediatric autoimmune diseases [316].

### 9.2. Role of Immunosuppressive and Immunomodulatory Therapy

Immunosuppressive agents, such as corticosteroids and more specific immunomodulatory therapies (i.e., biologics), and co-stimulatory blockers have been examined as tools for reducing the progression of the autoimmune process, especially in T1DM [319]. In addition, Teplizumab injections (an immunomodulatory agent approved for T1DM) may delay the onset of Stage 3 T1DM in patients with Stage 2 who have tested positive for two or more T1DM-related autoantibodies [319]. These agents could help manage pediatric autoimmune diseases like T1DM and AITD.

Teplizumab targets the CD3 receptor, delays the onset of T1DM in at-risk individuals, and delays the autoimmune process [273]. This is an important addition to prevent autoimmunity [320,321]. Targeting β-cells with other biologics, like rituximab, has also been investigated in autoimmune conditions [315]. They have successfully reduced disease flare-ups and enhanced remissions [273] and B-cell depletion [322].

Evidence suggests that preventive strategies may yield better outcomes than treatments in autoimmune diseases [323]. Early immune modulation using vitamin D, omega-3 fatty acids, and probiotics seems to reduce disease incidence and progression [315]. Studies on pre-symptomatic treatment with immune modulators like teplizumab [323] show that modifying immune responses before clinical onset can delay or prevent T1DM progression [274,314,324]. Early identification and treatment are the keys to managing these disorders, which is crucial for high-risk populations [315,323].

As a new strategy for overcoming T1DM in children, gene-based therapies are also being explored [325]. For example, the PROGRESS trial in adults evaluated KRIYA-839, an adenoviral vector engineered to express glucokinase and insulin, allowing the capsid and transgene to avoid the host immune response (by genetically modifying these components), as well as optimizing the large-scale production of AAV [326]. Parallel approaches continue to investigate islet cell transplantation without systemic immunosuppression [327], including the use of allogeneic UP421 primary islet cells [328].

### 9.3. Preventing Childhood Autoimmune Diseases Through Lifestyle, Nutritional, and Orthomolecular Intervention

Lifestyle modifications are essential to reduce most chronic diseases, including autoimmune diseases. Diet, physical activity, stress management, and controlling environmental factors could positively influence immune function and contribute to preventing the onset and/or exacerbation of autoimmune disorders [48]. Studies suggest that diets rich in anti-inflammatory foods and omega-3 fatty acids, like the Mediterranean diet, may reduce the risk of autoimmune diseases, including T1DM, RA, and MS [216,329].

Meanwhile, regular physical activity and good sleep help maintain physiological balance and support immune regulation, thus reducing autoimmune disease risk [330]. Chronic stress (thus elevated glucocorticoids) is known to disrupt immune function and heighten inflammation [48]. Therefore, managing stress without allowing it to accumulate is equally crucial. Pollution prevention [95] and mindfulness meditation, yoga, and relaxation techniques can also help reduce stress, strengthen immune health, and potentially lower autoimmune disease risk [331,332].

Environmental factors—such as reducing exposure to pollutants and preventing infections by supporting a strong immune system—can play an important role in lowering the risk of autoimmune disease. Therefore, adopting a healthy lifestyle that includes dietary improvements, nutrient-dense foods, regular exercise, and stress reduction [48], while minimizing contact with environmental toxins known to trigger autoimmunity, supports overall health and immune function [1,95], particularly in conjunction with early genetic screening and timely intervention [333].

To exploit extrarenal calcitriol synthesis, researchers have investigated the injection of CYP27B1-overexpressing dendritic cells or macrophages in vivo to increase local, targeted calcitriol production in peripheral lymphoid tissues. This approach stimulates CD4+ regulatory T cells and promotes stem cell differentiation, offering potential therapeutic applications for suppressing autoimmune diseases [10,334]. Despite this, gaps persist in our understanding of interactions between micronutrients and autoimmune diseases and the role of personalized nutrition in managing pediatric autoimmune disorders.

Advances in micronutrient research in immunological science could offer valuable insights for improved management. Exploring integrative medicine, functional foods, orthomolecular holistic approaches, and personalized nutrition may also present promising opportunities for future treatments at a lower cost [335,336]. Figure 6 illustrates the fundamental strategies that can be implemented to minimize childhood autoimmune disorders.

### 9.4. Recommendations of Vitamin D to Mitigate Childhood Autoimmune Diseases

Adequate nutrient intake, including calcium, protein, and micronutrients, is critical during childhood due to growth-related demands [147,337,338]. Since human breast milk has too little vitamin D (between 10 and 40 IU/liter), all breastfed infants should receive 600 IU/day of vitamin D supplementation [339]. The American Academy of Pediatrics Food and Nutrition Board [340,341] recommends 400 IU/day for infants under 12 months and 600 IU/day for children. Most guidelines, however, are based on the 2011 IoM report from 2011 [342]. As described in Section 3.1, this may no longer be accurate or current [167,168,169,343].

The above-mentioned statistical error in the IoM report made its vitamin D recommendations tenfold lower than intended [167]. Thus, the 2011 IoM-recommended dose of 600 IU/day should have been 6000 IU/day [167,168,169,343,344]. Instead of correcting it, this error was defended by the IoM authors [345]. Since then, the error has been perpetuated in several scientific society recommendations, including from the UK [346,347], the USA, NIH [348] and TES (2024) [187], and European Societies [187,349,350].

Beyond infancy, most government recommendations and clinical guidelines for vitamin D remain inadequate because they focus almost exclusively on bone health while largely ignoring its extra-skeletal benefits, including the immune system and mitigating autoimmune disorders [75,78,188]. In contrast, recent data support that the optimal function of extra-skeletal physiological systems requires substantially higher vitamin D intakes [12,78] and higher serum 25(OH)D concentrations than currently recommended [75,77,189,195,351,352].

Evidence indicates that maintaining a physiological serum 25(OH)D concentration above 40 ng/mL generally requires approximately 6000 IU/day for a 70 kg adult; not the government’s recommendation of 600 IU/day [163,167]. The former amount is necessary to activate the immune system and to prevent autoimmunity [351,352]. Consequently, current recommendations from the National Institutes of Health [348], the ES [187], and the UK National Institute for Health and Care Excellence (NICE) [353] are outdated and unhelpful, and require revision [78]. Extrapolating intakes and blood levels to prevent rickets in children [340,341] and osteomalacia in adults [163] to extra-skeletal systems and prevention of other diseases [75,77] is misleading in guidelines and clinical practice [185,354,355].

The minimum circulation of 25(OH)D level above 40 ng/mL, with an optimal range of 40 to 80 ng/mL, covers over 90% of the common disorders [77,78] irrespective of age [12,177]. Whereas concentrations exceeding 50 ng/mL are necessary to overcome infections, cancer, and autoimmune diseases and to lower all-cause mortality [68,172,173,356]. The range of 50 and 80 is estimated to cover 99.8% of human disorders [12,68,78]. New findings support the development of new evidence-based clinical guidelines for vitamin D intake and optimal serum 25(OH)D concentrations to maintain health and prevent or manage disease, including reducing risks for autoimmune diseases.

### 9.5. Future Research and Therapeutic Approaches

Future therapeutic strategies for pediatric autoimmune-related endocrine disorders need to focus on prevention rather than solely symptomatic treatments, as practiced currently [46]. As with most chronic diseases, lifelong, symptom-based treatments for these disorders are highly expensive and profitable; consequently, root-cause-based curative aspects are neglected. Considering this, physicians should focus on maintaining good health in children, improving overall nutrition and public health, and adopting holistic approaches that encompass vitamin D and cofactor sufficiency during pre-pregnancy, pregnancy, and lactation in mothers, infancy, and childhood in children, with balanced diets and daily physical activity, and ensuring micronutrient sufficiency and preventing harmful exposure [357]. Identifying genetically susceptible ethnic groups and implementing preventative actions are vital to achieving this goal.

Recently introduced immune modulators, such as teplizumab, are a promising approach to delaying the progression of T1DM in children [320,358]. Additionally, vitamin D supplementation has shown potential in preventing or slowing the progression of autoimmune destruction in pediatric autoimmune disorders, such as T1DM and autoimmune thyroiditis [126]. Other studies indicate that vitamin D may play a role in reducing the risk and preventing the onset of T1DM [274,359]. It is necessary to assess the costs, benefits, and ethical considerations of treating pre-symptomatic patients. These emerging therapies hold promise but will require careful examination in the context of long-term outcomes and ethical implications in pediatric care.

## 10. Prevention and Treatment of Childhood Autoimmune Disorders

Childhood autoimmune disorders are best managed by those with expertise in prevention, early recognition, prompt diagnosis, and providing individualized treatment. Those children with a strong genetic and immune basis need to be attended to with focused primary prevention. Once identified, treatment typically combines disease-specific or disease-modifying medications such as targeted immunosuppressive or anti-inflammatory therapies with supportive care, including nutrition, monitoring, pre- and probiotics, and management of complications to reduce long-term organ damage and improve quality of life, preferably in specialized centers.

### 10.1. Reducing Risks of Pediatric Autoimmune Disorders via Vitamin D Sufficiency

Vitamin D enhances intestinal defense mechanisms by regulating the mucosal immune system and suppressing harmful microbiota proliferation [360,361]. Vitamin D deficiency increases the risk of IBD, highlighting its importance in gut health. Individuals with hypovitaminosis D who develop IBD experience more severe disease with rapid progression and poorer outcomes, compared to those with sufficient vitamin D levels [129,130]. These findings underscore vitamin D’s critical role in gut–immune homeostasis and in mitigating inflammation [360,362].

Monitoring 25(OH)D levels (annually with other routine blood tests) helps with early identification and avoidance of severe vitamin D deficiencies, allowing rectification to reduce risks of immune dysfunction, infections, and autoimmune disorders [77,78,363]. It is especially vital for children with immune deficiencies due to other reasons, underlying comorbid conditions like obesity or immune compromise, as deficiency heightens susceptibility to infections and autoimmune diseases [77,78]. Proper supplementation supports innate and adaptive immune responses [12,177], reducing immune-related disorders and infections while improving long-term pediatric health [364]. Section 7 provides details of common pediatric autoimmune disorders.

### 10.2. Sun Exposure and Vitamin D Supplementation—Dose Recommendations

***Sun Exposure:*** For children/families, 30–45 min of direct daily sun exposure (depending on skin pigmentation), with approximately one-third of the upper body exposed and the eyes protected, is generally sufficient to produce adequate vitamin D [365]. Those with lighter skin can generate similar amounts with about half that exposure time. If remaining outdoors after sufficient sun exposure, it is advisable to apply sunscreen to reduce the risk of skin damage [203]. Vitamin D synthesis is reduced in most skin abnormalities, such as scarring, and with increased melanin content (i.e., darker skin), indirect sunlight, clothing, and sunscreen overuse [203]. In addition, certain medications, including anti-epileptics and antiretrovirals, accelerate vitamin D catabolism, as does obesity [32,71]. Malabsorption syndromes impair the intestinal absorption of vitamin D, including in children.

***Vitamin D Supplementation:*** In temperate climates, at least during winter, individuals should adjust vitamin D intake based on body weight or serum 25(OH)D levels [12,71]. Vulnerable people and those with comorbidities should take vitamin D supplements throughout the year. Unlike in musculoskeletal and metabolic disorders, in many extra-skeletal disorders, it is recommended to maintain serum 25(OH)D concentrations above 50 ng/mL, such as to mitigate infections and autoimmunity [12], and cancer [264,366,367], and improve all-cause mortality [32,68,75,368,369,370].

The daily dose can be calculated easily, based on the serum 25(OH)D concentration, BMI, or body weight [32,71] (see below). However, testing for 25(OH)D is costly and often inaccessible in developing countries. To address this, the senior author of this study developed tables based on many published datasets to calculate the precise amounts of vitamin D that individuals need to take to maintain serum 25(OH)D concentrations above 50 ng/mL [71]. Similar serum 25(OH)D levels can also be achieved with 1000 IU per 10 kg of body weight, but the variability can be high in this crude estimate, based on an individual’s metabolism, body fat, etc. These methods are provided as tables, cover daily and weekly vitamin D doses, as well as bolus doses, based on body weight and/or body mass index (BMI), as well as serum 25(OH)D concentrations when available.

The tables were subsequently simplified to a three-body-weight-categories/BMI formula with ranges [12] that would maintain serum 25(OH)D concentrations above 50 ng/mL [32]. These provide an easily calculable, flexible vitamin D range tailored to the needs of most individuals, designed to maintain serum 25(OH)D concentrations above 50 ng/mL, regardless of age, ethnicity, location of residence, or skin color [12,68].

   I. Not obese (average wt.: BMI < 29): 70–90 IU/kg BW
  II. Moderately obese (BMI 30–39): 100–130 IU/kg BW
III. Morbid obesity (BMI over 40): 140–180 IU/kg BW

Notably, circulating vitamin D concentrations are influenced by body weight, BMI, diet, sun exposure, supplemented dose, health status, concurrent medications, and genetic factors, necessitating individualized dosing to achieve the desired blood level. One dose will not fit all [77,78,176].

### 10.3. The Importance of Maintaining Therapeutic Levels in Preventing Autoimmunity

As many recent clinical studies have shown, maintaining serum 25(OH)D concentrations above 40 ng/mL (preferably >50 ng/mL), with the optimal range being 50–80 ng/mL, enhances immune functions, reduces hospitalizations and mortality, and is likely to substantially lower healthcare costs. In non-obese children, approximately 70 IU/kg/day of vitamin D_3_ generally maintains serum 25(OH)D concentrations within the 50–80 ng/mL range, whereas obese children require considerably higher doses, as indicated in the above formulae [32,71]. These safe, cost-effective regimens support disease prevention and are likely to improve clinical outcomes [75,77].

When a given community has widespread vitamin D deficiency, a targeted staple food fortification program is an economical way to provide vitamin D [371,372] alone or in combination with additional targeted cofactors [373,374]. Meta-analyses have confirmed that vitamin D fortification can increase serum 25(OH)D concentrations by about 6–10 ng/mL (15–25 nmol/L); however, individual responses vary significantly due to food habits and partly because of the nonlinear dose responses [375].

Modeling suggests that if all 34 European countries implemented sufficient vitamin D food fortification, approximately 129,000 cancer deaths (about 9% of all cancer deaths) could be prevented annually. This assumes that fortification increases 25(OH)D levels sufficiently to reduce cancer mortality. It highlights the importance, practicality, and economic benefits of food fortification programs [371,376]. Another option is to encourage daily, sufficient, safe sun exposure [377].

Along with adequate vitamin D intake, lifestyle modifications can help reduce the risk of autoimmune diseases in children. A balanced diet rich in anti-inflammatory foods, including fruits, colorful vegetables, and omega-3 fatty acids, supports immune health and is recommended [338,378]. In addition, regular physical activity, adequate sleep, and effective stress management through practices such as mindfulness meditation or yoga further promote overall well-being and may reduce the likelihood of autoimmune dysregulation [379].

### 10.4. Optimizing Micronutrient Status to Reduce the Risk of Childhood Autoimmune Diseases

Adequate micronutrient intake throughout childhood is vital for robust immune development, which may reduce the risk of autoimmune disorders. While a balanced diet is ideal, supplementation may be necessary for deficiencies and/or increased physiological and growth demands. This should include proper micronutrient interventions with vitamins, antioxidants, and minerals [90] essential to enhance immune tolerance [380], which lowers autoimmune disease risk [378]. However, this is rarely accomplished by one supplement. Since micronutrients are threshold compounds, excessive supplementation is unlikely to provide added benefits [379]. Similarly, they are network nutrients, and so require multiple cofactors to obtain the optimum benefit [90]. Since the quantities of micronutrients necessary for managing autoimmunity are discussed in other sources, they are not covered here [313]. Instead, key micronutrients essential for immune function and minimizing autoimmune risks are summarized below.

***Vitamin D*** plays a pivotal role in modulating the immune response. As discussed above, over two-thirds of immune functions rely on vitamin D sufficiency [68,177]. Consequently, deficiency in vitamin D has been associated with an increased risk of autoimmune diseases such as T1DM. Supplementation with vitamin D (Section 8.2), as recommended above, is known to reduce chronic inflammation and the risk of autoimmune diseases, which enhances the robustness of the immune system [379].

***Omega-3 Fatty Acids*** are known for their anti-inflammatory properties, especially higher omega-3 to omega-6 ratios [127]. They positively modulate immune regulation. Incorporating omega-3-rich foods and supplements is recommended to positively modify immune responses and reduce the risk of autoimmune conditions [378,379].

***Zinc*** is essential for immune function. Zinc deficiency impairs immune responses and facilitates viral entry into human cells [381]. Along with other micronutrients like selenium, zinc sufficiency protects epithelial barriers and prevents viral entry into human cells (as with SARS-CoV-2) and fluid leaks. Sufficient levels are necessary to maintain immune homeostasis and tolerance [380] and prevent autoimmune reactions [378].

***Probiotics***: Gut microbiota play a significant role in immune system development [382,383]. Therefore, probiotic supplementation can help maintain a healthy gut flora balance, which is crucial for immune regulation and prevents autoimmune diseases [379,380].

### 10.5. Adverse Effects―Avoidance of Excess Use of Micronutrients

While supplementation of targeted micronutrients can be beneficial, excessive intake of certain micronutrients should be avoided to minimize unnecessary cost and potential adverse effects. Therefore, they should be tailored to individual needs and deficiency states, provided under the guidance of a healthcare professional, including pediatric dietitians. Adverse effects from vitamin D are very rare; when they occur, they are mostly due to mistakenly taking extremely high doses.

Vitamin D toxicity, such as hypercalcemia and hypercalciuria, could manifest when serum 25(OH)D concentration exceeds 150 ng/mL (>375 nmol/L) [32,71,384]. Most reported cases are due to mistaken or inappropriate doses taken for prolonged periods [385,386,387,388,389,390,391]. Chronic intake of vitamin D exceeding 10,000 IU/day in non-obese individuals consuming a normal- or high-calcium diet may lead to vitamin D toxicity, or those concurrently taking thiazides or lithium may also present with high serum 25(OH)D concentrations [83,84].

Signs and symptoms of vitamin D toxicity are primarily due to hypercalcemia and hypercalciuria. Manifestations of hypercalcemia include loss of appetite, weight loss, nausea, vomiting, constipation, weakness, fatigue, confusion, cognitive impairment, cardiac arrhythmias, and, in severe untreated cases, death [387,389]. Hypercalciuria may cause polyuria, polydipsia, dehydration, and an increased risk of nephrolithiasis.

If vitamin D toxicity is suspected, vitamin D and calcium supplementation should be stopped immediately, and the patient’s history reviewed for duplicate sources of either supplement. Prompt rehydration resolves most cases; temporary dialysis is rarely required. Given its high clinical value, excellent cost-effectiveness, and favorable safety profile at recommended doses, vitamin D supplementation should be used to maintain serum 25(OH)D concentrations between 50 and 80 ng/mL [71,76]. It is recommended as a routine in pediatric care to minimize symptomatic infections, complications, and the development of immune-related problems.

At high doses, vitamin D may interact with medications that are metabolized by cytochrome P450 3A4 *(CYP3A4)* [212,214]; it can also affect the actions of anticonvulsants, antiretroviral agents, statins, thiazide diuretics, verapamil, and digitalis preparations [68]. However, in most cases, in hospitalized patients, the most common cause of hypercalcemia is an incidental laboratory finding, and it is related to cancer due to the inappropriate secretion of parathyroid hormone-related peptide (PTHrP) or metastatic bone disease [83,84].

Further research may be able to establish definitive recommendations for specific micronutrient supplementation in preventing autoimmune disorders and promoting children’s health [378]. Figure 7 summarizes a commonsense approach to the management of vitamin D deficiency in the pediatric population.

### 10.6. Maintaining Optimal Health Through Balanced Micronutrient and Mineral Intake

Minerals are vital for physiological functions [90], including bone health, enzymatic reactions, nerve signaling, and immune regulation [392]. Deficiencies in macro minerals like calcium, magnesium, and phosphate can lead to osteoporosis [90], cardiovascular issues, and immune dysfunction [393] and neuromuscular disorders [394,395]. Similarly, as discussed above, inadequate iron, zinc, and selenium intake (Table 1, Section 2.2) can impair oxygen transport, weaken immunity, delay wound healing, and increase inflammation and susceptibility to infections and autoimmune diseases [396]. Selenium deficiency also elevates inflammatory cytokines via the iNOS/NF-kB pathway [393,397]. A well-balanced diet, targeted supplementation, and public health initiatives are essential to preventing long-term health consequences [90].

On the other hand, excessive mineral intake can have serious adverse effects [90]. For instance, hypercalcemia, often due to cancer and sometimes excessive calcium intake (and exceptionally rarely with prolonged mega-doses of vitamin D), can lead to kidney stones and cardiovascular abnormalities [90] and neurological dysfunction [392]. Similarly, excessive iron levels can cause oxidative stress and tissue depositions (hemochromatosis), leading to tissue and organ damage [394], particularly affecting the liver, testes, pancreas, and heart, and weakening the immune system, increasing autoimmunity risks [393].

The above-mentioned findings emphasize the importance of maintaining mineral intake within an optimal range to prevent both deficiencies and toxicities [90]. Figure 8 summarizes the importance of maintaining a balance between immune and endocrine systems, and associated cofactors in conjunction with vitamin D status in avoiding autoimmune diseases in childhood.

Dietary diversity, safe sun exposure, responsible supplementation, and population-specific public health policies are necessary to achieve balanced vitamin and mineral intake. Educational initiatives and food fortification programs can be used complementarily to help mitigate micronutrient imbalances, especially in vulnerable groups such as children [374,393]. Additionally, individualized assessments based on children’s dietary habits and genetic predispositions can provide more precise mineral intake recommendations. These approaches can reduce the risk of both deficiency-related disorders and toxicity. Ensuring appropriate mineral consumption is key to promoting overall health and preventing chronic diseases in children [90].

## 11. Discussion

Micronutrients, particularly vitamin D and associated cofactors, are essential for maintaining endocrine and immune system health. These nutrients regulate immune responses, support hormonal balance, and enhance metabolic pathways. Deficiencies, especially in vitamin D and magnesium, can weaken immunity, exacerbate autoimmune diseases, and disrupt endocrine function. Understanding the interactions between micronutrients, the immune system, and endocrine pathways is crucial for early intervention, therapeutic advancements, and improved health outcomes in children.

Key vitamins such as D, A, C, and E, as well as minerals like zinc, iron, and selenium, play significant roles in immune and endocrine regulation [90]. Vitamin D modulates immunity by regulating T-cells, and pro-inflammatory cytokines are critical in autoimmune diseases like T1DM and AITD [251,398]. Maintaining adequate 25(OH)D levels reduce autoimmune disease risk, emphasizing the pivotal role of micronutrients in immune and endocrine health [50,176].

Modern medicine should recognize the value of nutrients and nutraceuticals, which offer more cost-effective natural alternatives with fewer adverse effects for disease prevention. Additionally, many individuals either lack access to or cannot afford fresh, nutritious fruits and vegetables, making it challenging to maintain a balanced diet and a healthy lifestyle that includes proper exercise. Most health issues, including autoimmune disorders, can be improved with proper education, affordable nutritious food, and targeted supplements. It is time to integrate holistic and orthomolecular medicine into mainstream medicine.

The impact of micronutrients extends beyond simple nutritional support, as they play complex roles in regulating the immune system and endocrine function, often acting in concert to prevent or mitigate autoimmune diseases in children [399]. For example, zinc and selenium regulate thyroid hormone metabolism and immune system balance, with deficiencies linked to an increased risk of AITDs and impaired immune function. Additionally, retinoic acid and vitamin A also influence immune responses through their active form. It helps regulate gene expression in immunity, potentially protecting against developing autoimmune disorders [400].

The rising incidence of autoimmune diseases in children underscores the need for population-based strategies to mitigate their impact [46]. In addition to nutrient supplementation, identifying and minimizing environmental triggers is crucial for reducing risks in pediatric populations [401]. Expanding public health initiatives to educate pediatricians, nurses, and parents on autoimmune diseases, mechanisms, and risk factors will enhance prevention efforts and improve child health.

Vitamin D supplementation reduces the incidence of autoimmune diseases in children and slows the progression of conditions such as T1DM and AITD [126]. Similarly, omega-3 fatty acids, which modulate immune [73] and endocrine functions, show promise by reducing inflammation and promoting immune tolerance [50,399]. However, awareness among healthcare professionals and the public remains limited. Further hypothesis-driven research is needed to define synergistic interactions and optimize the personalized use of micronutrients to modulate immune and endocrine function in children [50].

The increase in micronutrient deficiencies that impair endocrine and immune function in children with autoimmune diseases highlights the need to optimize nutrition and minimize food additives and preservatives in clinical practice. By promoting appropriate micronutrient supplementation and healthy lifestyle changes, informed healthcare providers can improve health outcomes and slow disease progression. Continued advances in this field will strengthen immune and endocrine health, reduce infections, mitigate autoimmune disorders, and improve long-term outcomes in children.

Vitamin D is the most important micronutrient for immune function and understanding its interaction with the endocrine and immune systems is essential for protecting vulnerable children. Vitamin D deficiency causes significant immune dysfunction, increasing the risk of preventable diseases. Parents, teachers, school administrators, and healthcare professionals should be educated about the importance of safe sun exposure and maintaining adequate 25(OH)D levels through diet, supplementation, food fortification, and healthy lifestyle practices, so that they can help children better. Translating this knowledge into clinical practice and public health recommendations is essential to improve children’s health outcomes.

## 12. Conclusions

Pediatric autoimmune disorders are major chronic diseases resulting from immune dysregulation that can affect multiple organs, particularly the endocrine and immune systems. Growing evidence indicates that deficiencies of essential micronutrients—including vitamin D, zinc, selenium, and omega-3 fatty acids—contribute to immune dysfunction and increase susceptibility to autoimmune diseases, especially in genetically predisposed or immunocompromised children. Therefore, early identification and correction of these deficiencies may help restore immune balance, improve endocrine–immune physiological interactions, reduce disease severity, and improve long-term clinical outcomes. This review highlights the complex interplay between micronutrients, the endocrine system, and immune function, with particular emphasis on the nutrient vitamin D. It underscores the need for further research to clarify the underlying mechanisms and evaluate targeted nutritional interventions as effective adjuncts for preventing and managing pediatric autoimmune disorders.

## Figures and Tables

**Figure 1:**
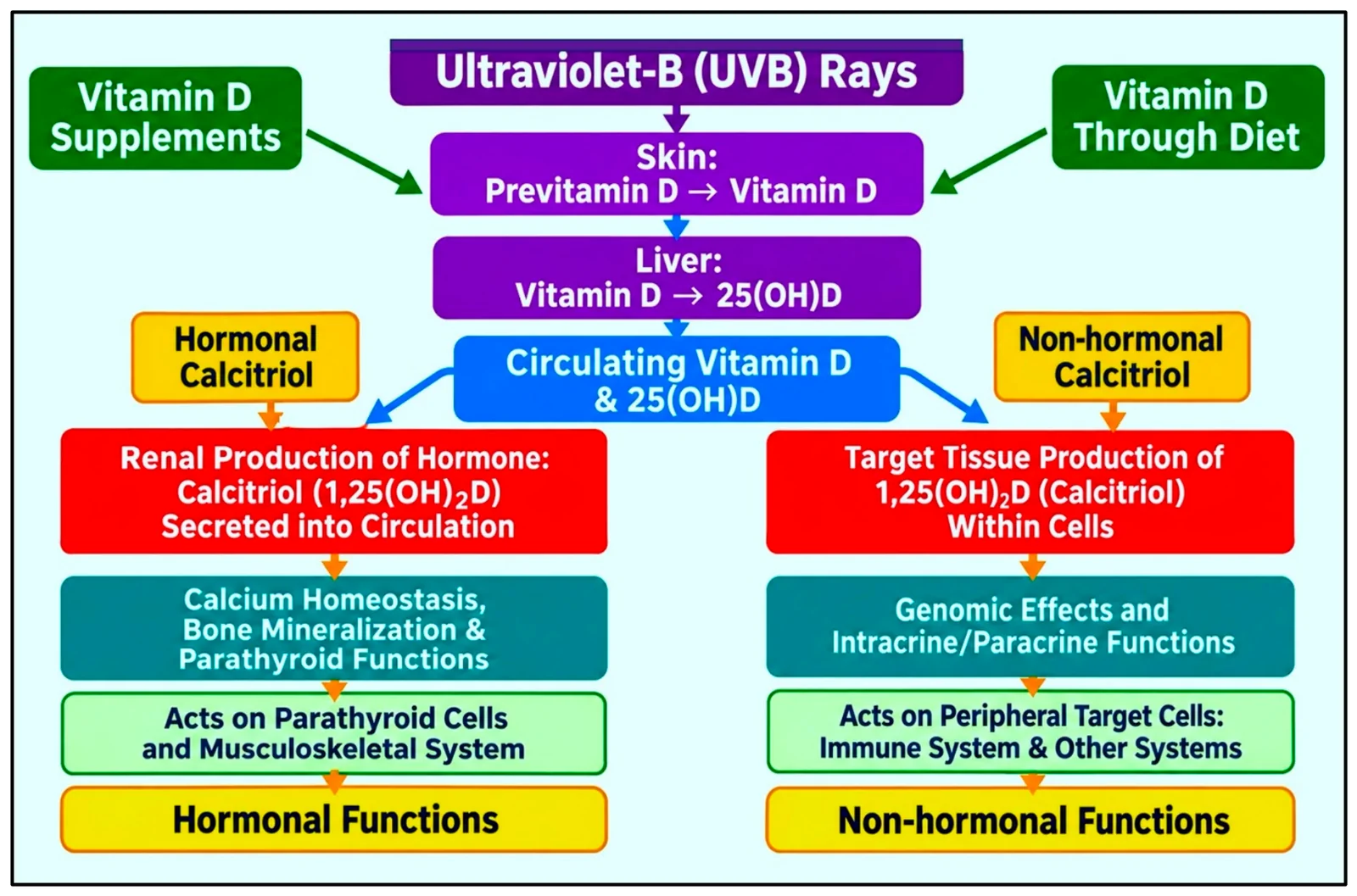
The vitamin D synthesis pathways, its hormonal and non-hormonal actions, and intracrine, paracrine, and genomic actions of vitamin D. A functional endocrine system is essential for maintaining homeostatic bodily functions and appropriate, rapid biological and physiological responses. Notably, micronutrients, vitamins, minerals, and other cofactors are indispensable for the proper functioning of the endocrine and immune system. Vitamin D metabolites profoundly modulate immune functions to facilitate defense against infections [11] and prevent autoimmunity [12]. These micronutrients modulate and support biochemical pathways and hormone release and their receptor interactions (e.g., calcitriol and its receptors; VDR (vitamin D receptor = calcitriol receptor = CTR)).

**Figure 2:**
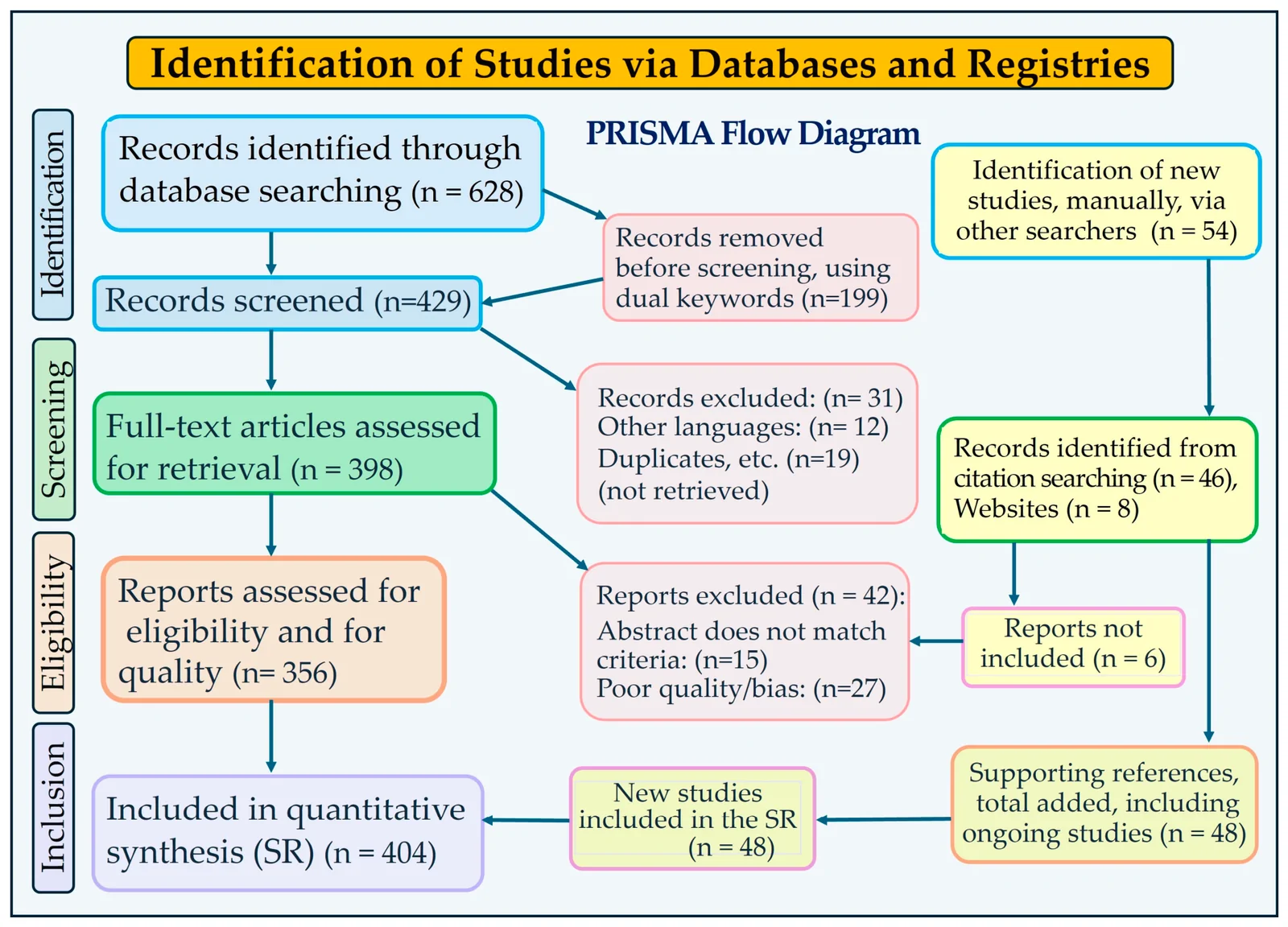
PRISMA flow chart—updated systematic searches of databases, identification, screening, and selection of research papers in this review (SR = systematic review, *n* = number of manuscripts).

**Figure 3:**
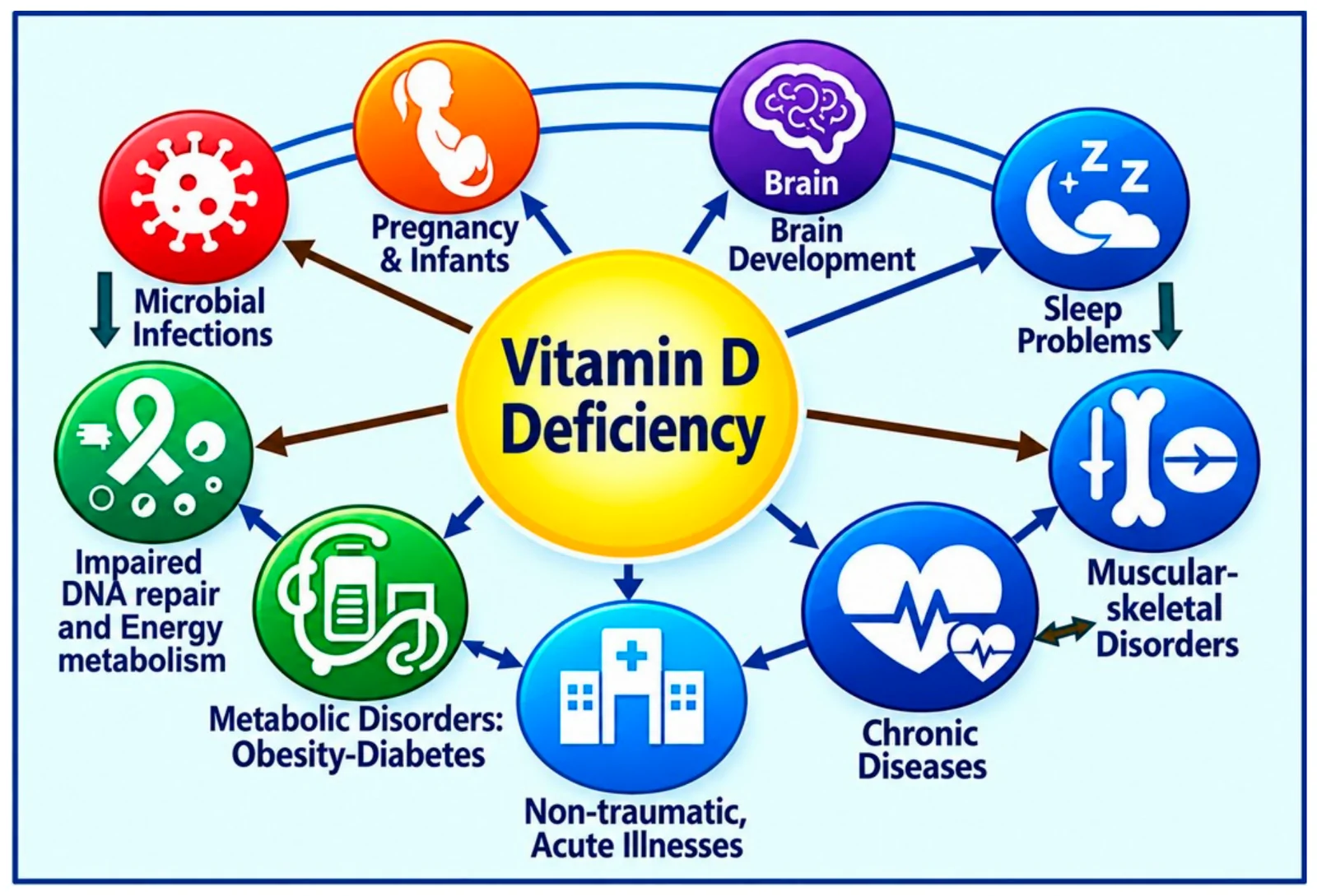
Major negative consequences of vitamin D deficiency are categorized into groups of physiological status and chronic disorders.

**Figure 4:**
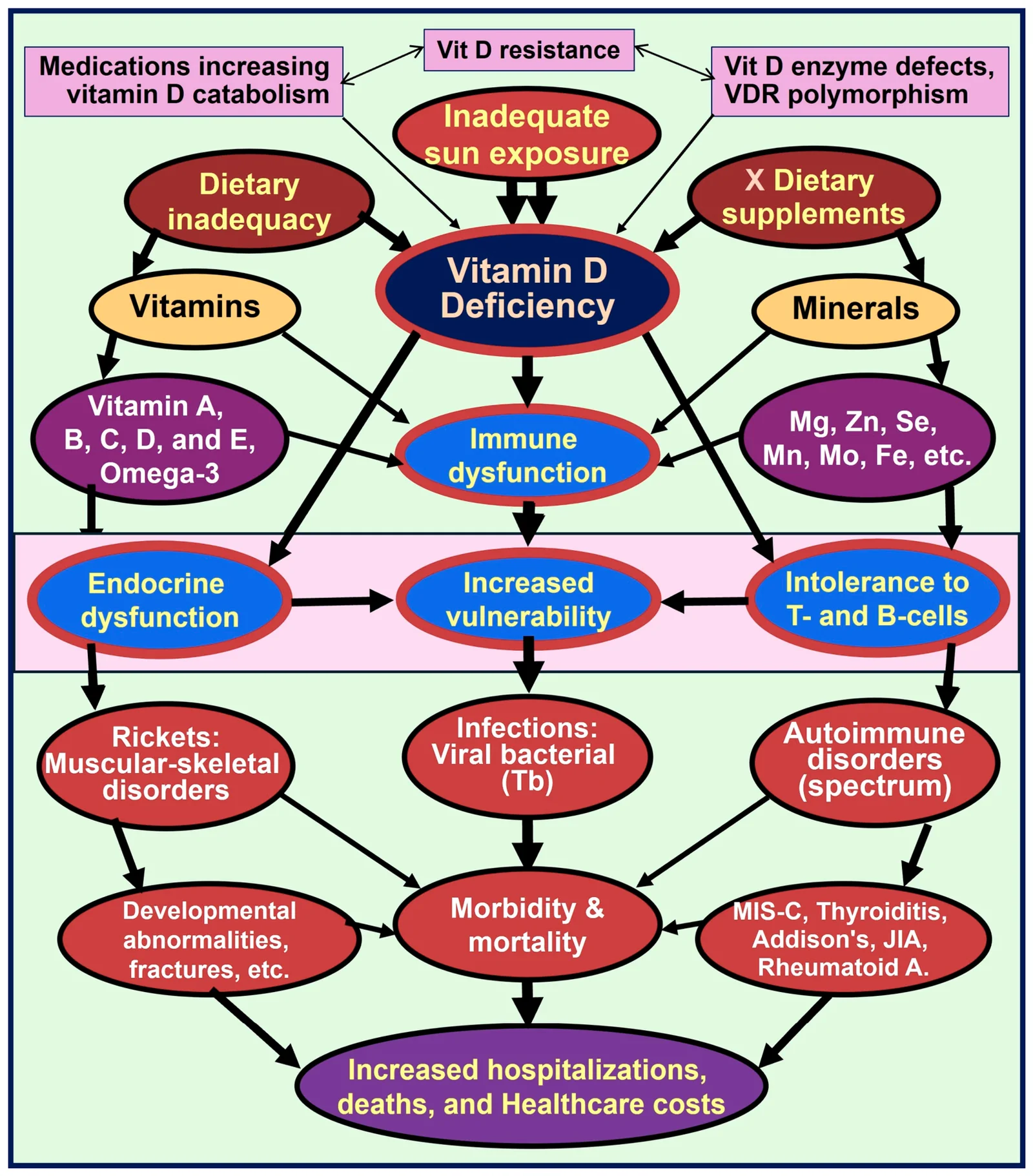
The top portion of the chart illustrates vitamin D synthesis, its interaction with essential cofactors like magnesium, and its pivotal role in maintaining overall health. The blue ovals in a light pink background in the middle section indicate clinical outcomes from hypovitaminosis D, which lead to endocrine and immune cell dysfunctions, contributing to disorders and increasing vulnerabilities to autoimmune diseases that are highlighted in red circles (bottom section). This interplay demonstrates the potential exacerbation of disease burdens, increases complications, hospitalizations, and mortality, and significantly escalates healthcare costs (JIA = Juvenile inflammatory arthritis; Rheumatoid A = Rheumatoid arthritis).

**Figure 5:**
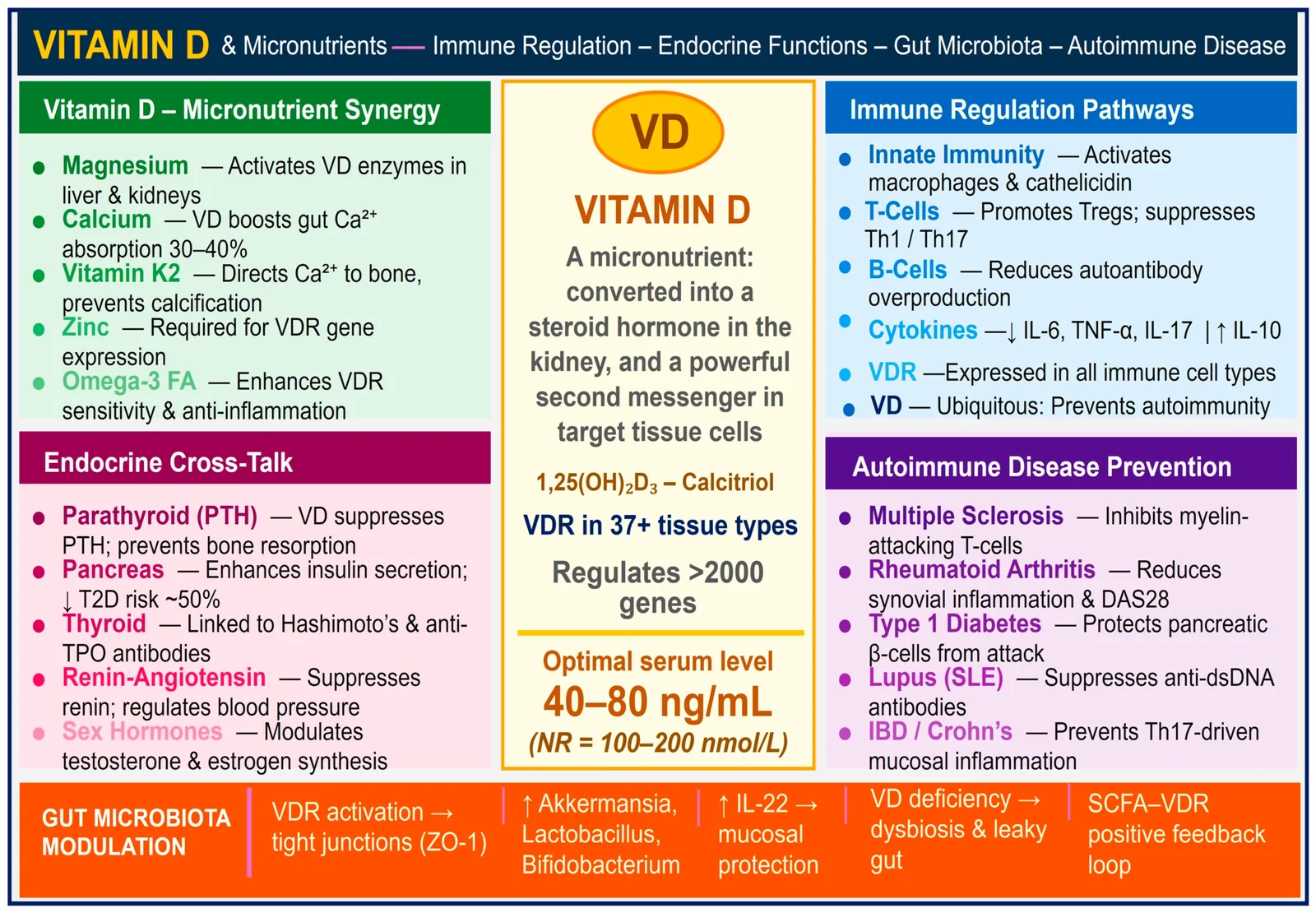
Illustrates multiple micronutrients and cofactors that influence endocrine system cross-interactions with the immune system. In the deficiency of vitamin D, the breakdown of this harmony leads to the development of various pediatric autoimmune disorders. ↑ Increase; ↓ decrease.

**Figure 6:**
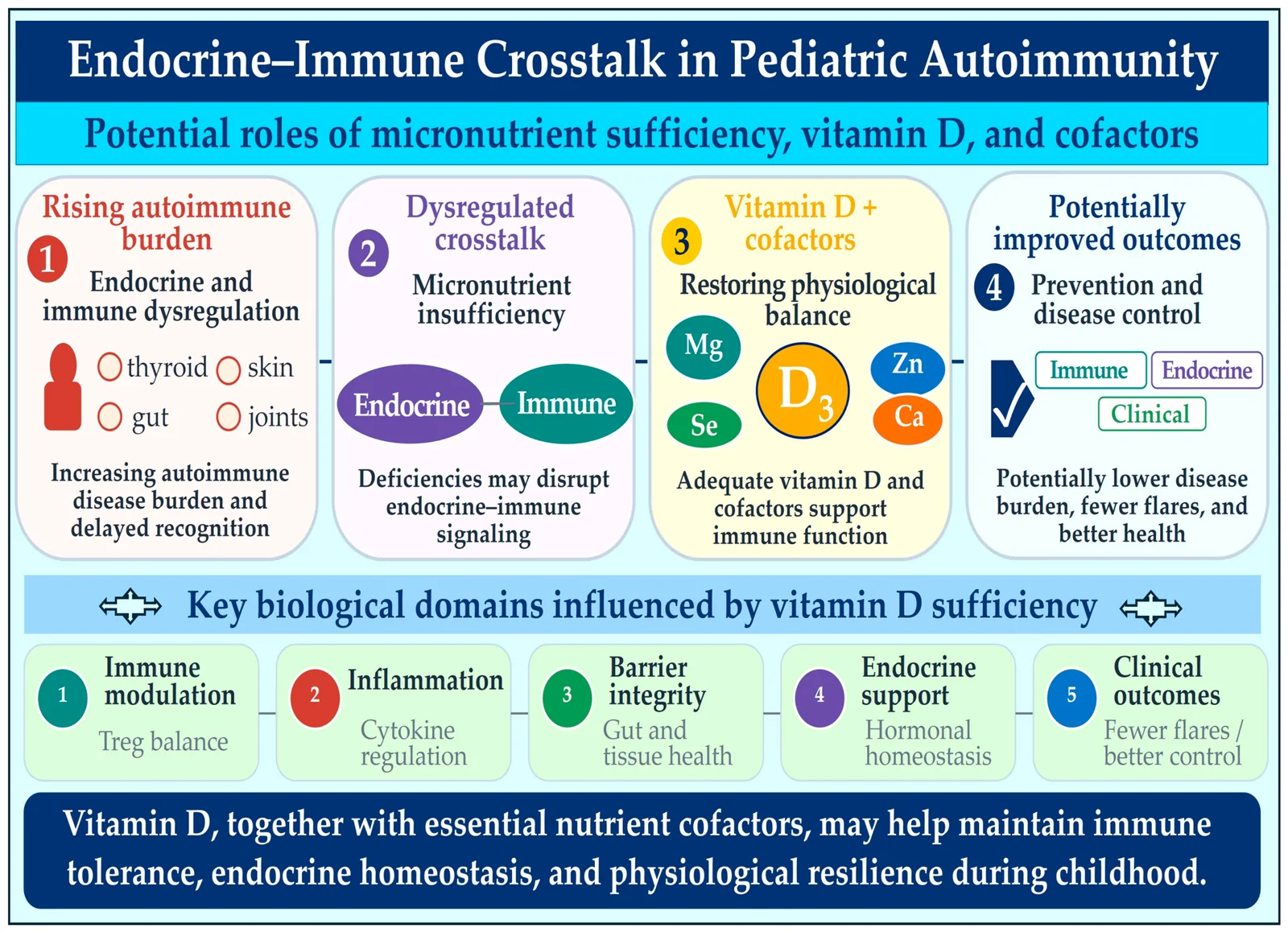
Integrative micronutrient strategies to strengthen immune resilience and endocrine harmony—a cost-effective approach to pediatric autoimmune prevention.

**Figure 7:**
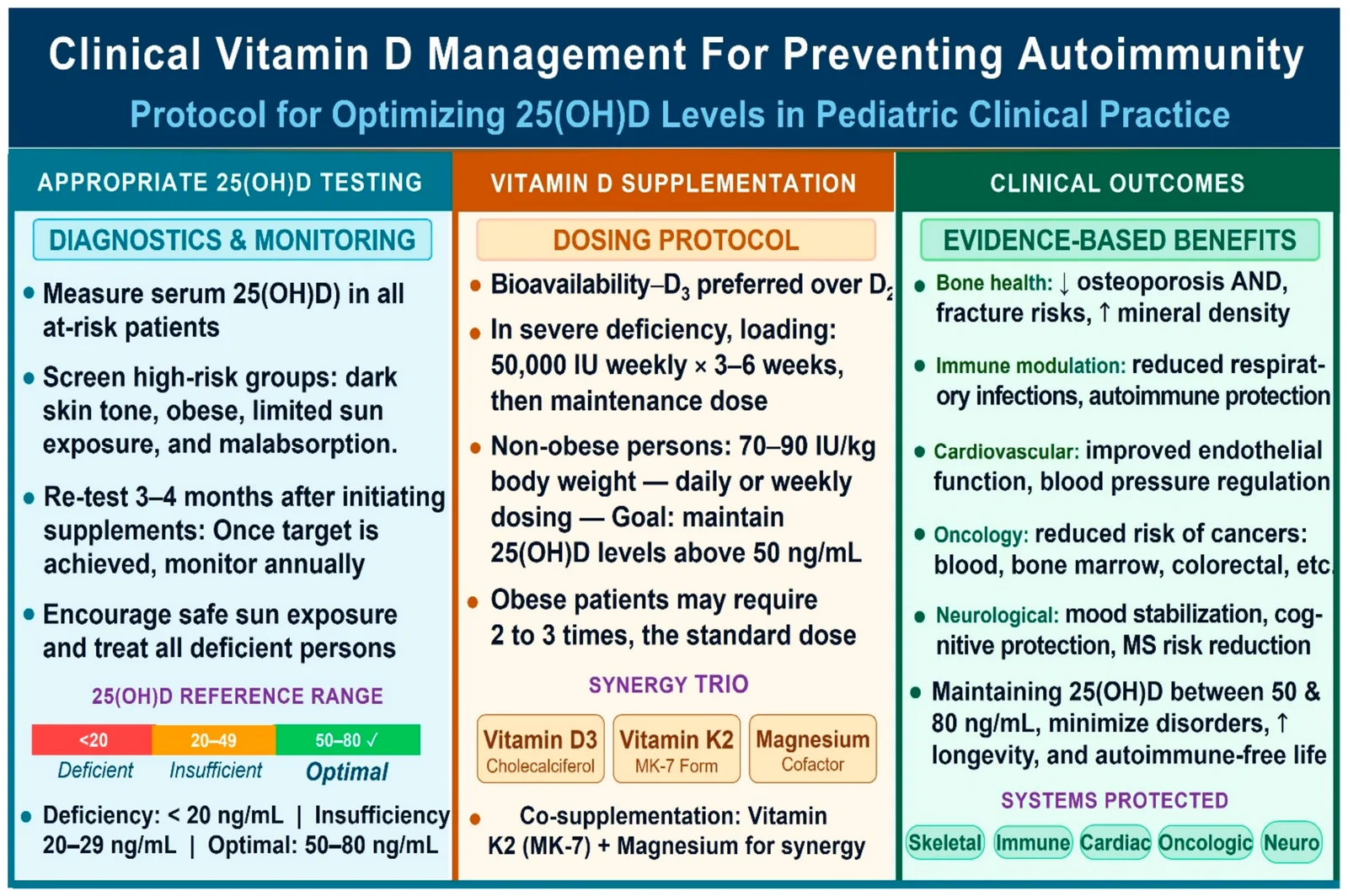
Strategy for optimization of vitamin D status in clinical practice in the pediatric population. Diagnosis and monitoring of vitamin D status, optimal supplementation methodology, and expected clinical outcomes. ↑ Increase; ↓ decrease.

**Figure 8:**
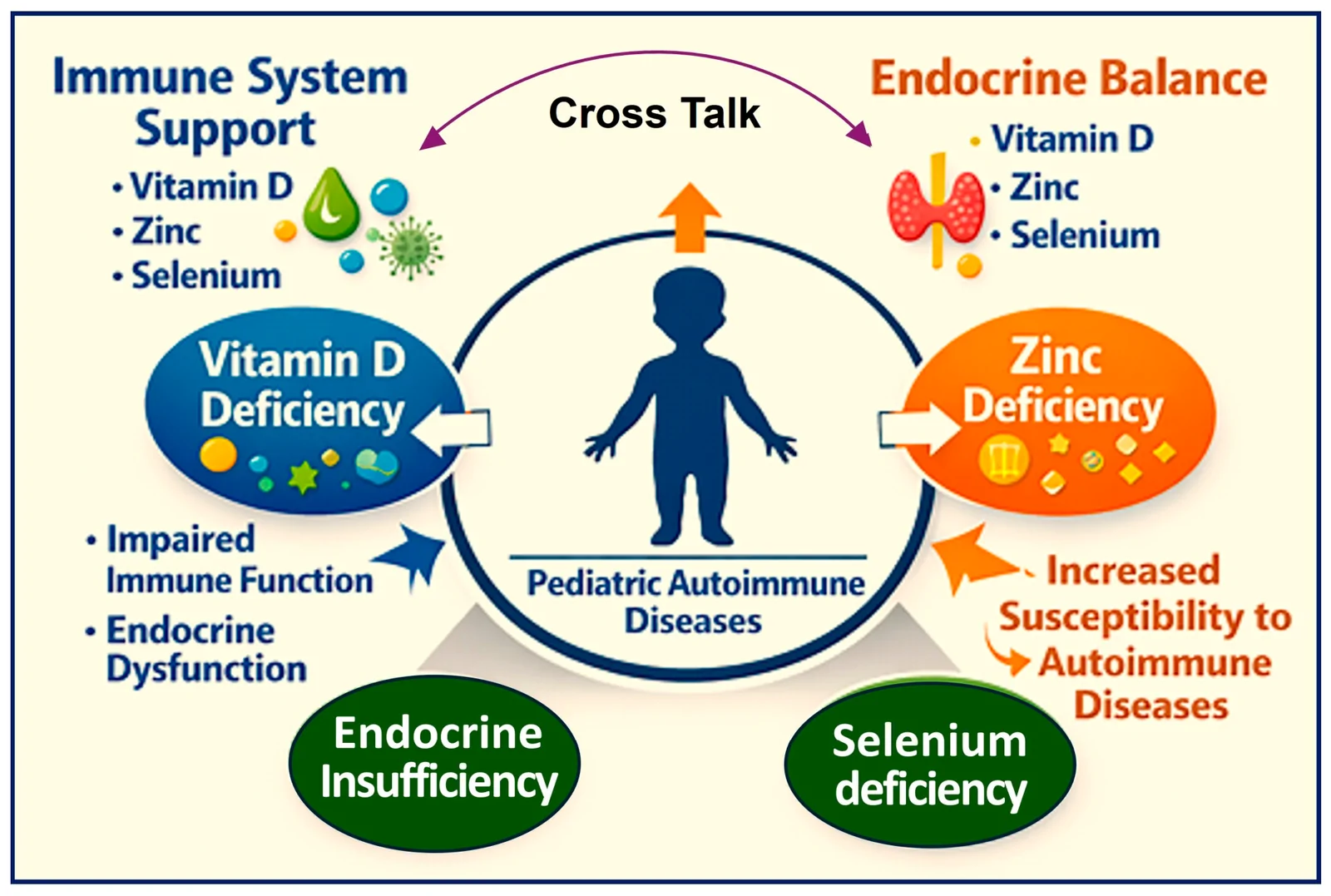
This integrated model illustrates the roles of vitamin D, its cofactors, and trace minerals such as zinc and selenium in maintaining immune function and endocrine balance. It also depicts how inadequate micronutrient status may impair immune–endocrine communication, contributing to dysregulated immune responses and greater susceptibility to pediatric autoimmune diseases.

**Table 1:** PICOS: Participants; Intervention; Comparison; Outcome elements; and Study design philosophies.

	PICOS Criteria	Conditions
1	Participants	Children up to age 18; having a family history, personal risks, or a diagnosis of an autoimmune disorder
2	Intervention	None; Vitamin D; solar UVB radiation; micronutrients; omega-3 fatty acids
3	Comparison/control	Retrospective, case-report, observational, epidemiological, community-based/ecological, longer-term follow-up studies, and randomized controlled studies
4	Outcome elements	Morbidity, complications, overall health, and death Relationship of serum 25-hydroxyvitamin D [25(OH)D] and incidence and prevalence of autoimmune diseases; changes in prevalence following micronutrient interventions.
5	Study design philosophies	Clinical trials–randomized controlled clinical trials; non-randomized studies, longer-term prospective larger observational clinical studies; non-randomized non-controlled trials.

**Table 2:** Interactions of vitamins and minerals on the immune system.

Vitamins and Cofactors	Vitamins and Minerals on the Immune System	Reference
Vitamin A	Plays a vital role in maintaining mucosal immunity, supporting epithelial integrity, and regulating the immune system’s response to pathogens. It also aids in differentiating T-cells and is essential for producing immune mediators like cytokines.	[97,98]
Vitamin B	This group of vitamins, particularly B6, B9 (folate), and B12, is critical for enzymatic reactions that support immune cell function, production, and differentiation. These vitamins and cofactors are necessary in DNA synthesis and are essential for cellular energy metabolism and immune responses.	[99,100,101]
Vitamin C	Vitamin C is known for its antioxidant properties and protects immune cells from oxidative stress. It also enhances the function of neutrophils, macrophages, and T-cells, and it helps maintain the integrity of the skin and mucosal barriers.	[102,103]
Vitamin D	Influences immune detection and surveillance by modulating T-cell function, enhancing pathogen recognition, and maintaining immune tolerance. Deficiency in vitamin D is known to be associated with increased risk of autoimmune diseases.	[12,104]
Vitamin E	Acts as an antioxidant that modulates oxidative stress and inflammation. It enhances immune cell function, including the proliferation of T-cells and the activity of natural killer cells.	[105]
**Minerals**		
Magnesium (Mg)	Mg functions as a cofactor in various enzymes and receptor functions: it is essential for releasing hormones from endocrine cells, CTR functions, and immune cell activity, and it helps regulate inflammation. Magnesium is also necessary for maintaining a healthy immune system.	[17,68,93,106,107,108]
Zinc (Zn)	Essential for developing, activating, and functioning immune cells, such as neutrophils and T-cells. Zinc is key in regulating immune responses, inflammatory processes, apoptosis in immune cells, and proper immune response against infections.	[17,91,109,110,111]
Selenium (Se)	Acts as a cofactor for antioxidant enzymes and regulates immune responses, particularly by promoting the activity of T-cells and macrophages. Selenium also supports thyroid function, which is integral to immune regulation.	[84,112,113]
Iron (Fe)	Necessary for immune cell proliferation and function, particularly for generating reactive oxygen species in pathogen killing. Iron deficiency can impair immune responses, while excess iron can promote inflammation and oxidative stress.	[17,114,115]
Manganese (Mn)	Functions as a cofactor in various enzymes necessary for immune cell activity; Mn regulates inflammation. It is also involved in brain health and mitigating autoimmunity in childhood.	[17,84,116]
Boron	Improves cognitive performance and brain functions, as well as antioxidant functions. In addition, boron modulates endocrine functions.	[117,118]
Copper	Copper is essential for enzyme functions and iron metabolism. In addition, it prevents brain function decline. It prevents demyelination and autoimmune reactions. However, excess has the opposite effect and reduces endocrine functions.	[84,119]
Iodine	Excess iodine can induce thyroid autoimmunity. This relates to TgAb; unmasking Tg epitopes triggers autoimmunity. However, low iodine intake causes iodine deficiency and leads to an increased incidence of endemic goiter and hypothyroidism.	[46,120,121]
Silicon	Long-term overexposure increases the risk of autoimmune diseases, especially rheumatoid arthritis.	[122,123]
Other trace elements	Lithium (Li), cadmium (Cd) and molybdenum (Mo), etc.	[17,106,124]

**Table 3:** Non-musculoskeletal effects of vitamin D *.

Improve and boost innate and adaptive immune systems—control infections, immune tolerance, and autoimmunity
Reduce autoimmunity (e.g., multiple sclerosis, T1DM, asthma) and neurological disorders, and enhance immune tolerance
Suppress inflammation, excessive oxidative stress, and tissue fibrosis
Reduce the incidence and severity of metabolic disorders—type 1 and type 2 diabetes, obesity, immune-mediated bone diseases like rheumatoid and lupus arthritis, and osteoporosis
Prevention of chronic diseases like certain types of cancers
Decreased incidence and severity of cardiovascular diseases and reduced all-cause mortality
Improve epithelial, endothelial, and blood–brain barriers and gap junctions
Reduce risks of diseases and complications related to pregnancy, fetus, and childhood
Protect and repair DNA and mitochondria and enhance energy generation
Improves quality of life and extends health span
Decreased pulmonary and renal-related morbidities and improved survival
Fewer developmental and degenerative brain disorders

Data synthesized from Wimalawansa, S.J., 2012 [54] * and from multiple other sources [63,164,178,179].

**Table 4:** Common examples of pediatric autoimmune diseases.

Key Conditions	Description	Reference
Type 1 Diabetes Mellitus (T1DM)	Type 1 diabetes mellitus (T1DM) results from autoimmune destruction of insulin-producing pancreatic β-cells, leading to insulin deficiency and hyperglycemia. The disease is primarily mediated by T cells, with genetic susceptibility and environmental factors contributing to its onset. Autoantibodies against β-cell antigens often precede clinical disease and serve as biomarkers of autoimmune activity, reflecting the combined roles of cellular and humoral immunity in disease progression.	[36,273,274,281]
Juvenile Idiopathic Arthritis (JIA)	JIA is characterized by persistent joint inflammation in children. Autoimmunity is critical to T-cell activation and cytokine dysregulation, particularly involving interleukin-6 (IL-6) and tumor necrosis factor-alpha (TNF-α). Current treatments target these pathways to reduce inflammation and prevent joint damage.	[46,275,282]
Autoimmune Thyroid Disease (AITD)	This group includes Hashimoto’s thyroiditis and Graves’ disease, where the immune system erroneously targets thyroid antigens. Studies highlight the importance of genetic susceptibility (e.g., HLA alleles) and environmental triggers of AITDs.	[36,276,277,283]
Celiac Disease	This condition is driven by an immune-mediated reaction to gluten, leading to small intestinal damage. The presence of HLA-DQ2 or HLA-DQ8 genes significantly increases susceptibility. Gluten exposure triggers an abnormal T-cell response, leading to villous atrophy in the intestine.	[275,278]

## Data Availability

The original contributions presented in this study are included in the article. Further inquiries can be directed at the corresponding author.

## Abbreviations

1,25(OH)_2_D	1,25-dihydroxyvitamin D
25(OH)D	25-hydroxyvitamin D
ANA	Anti-nuclear antibodies
AITD	Autoimmune thyroid disease
GD	Graves’ disease
HLA	Human leukocyte antigen
HPA	Hypothalamic–pituitary–adrenal
IU	International Units
IBD	Inflammatory bowel disease
JIA	Juvenile idiopathic arthritis
MS	Multiple sclerosis
MIS-C	Multi-system inflammatory syndrome in children
RCTs	Randomized controlled trials
RF	Rheumatoid factor
SLE	Systemic lupus erythematosus
SR	Systematic reviews
T1DM	Type 1 Diabetes Mellitus
TLR	Toll-like receptor
TPoA	Thyroperoxidase antibodies
UVB	Ultraviolet-B
VDR/CTR	Vitamin D (calcitriol) receptor
